# Nested Association Mapping of Stem Rust Resistance in Wheat Using Genotyping by Sequencing

**DOI:** 10.1371/journal.pone.0155760

**Published:** 2016-05-17

**Authors:** Prabin Bajgain, Matthew N. Rouse, Toi J. Tsilo, Godwin K. Macharia, Sridhar Bhavani, Yue Jin, James A. Anderson

**Affiliations:** 1 Department of Agronomy, Purdue University, 915 West State Street, West Lafayette, IN 47907, United States of America; 2 Department of Agronomy and Plant Genetics, University of Minnesota, St Paul, MN 55108, United States of America; 3 United States Department of Agriculture-Agricultural Research Service (USDA-ARS), Cereal Disease Laboratory, St. Paul, MN 55108, United States of America; 4 Department of Plant Pathology, University of Minnesota, St. Paul, MN 55108, United States of America; 5 Agricultural Research Council – Small Grain Institute, Bethlehem, 9700, Free State, South Africa; 6 Kenya Agricultural and Livestock Research Organization (KALRO), Njoro, Kenya; 7 International Maize and Wheat Improvement Center (CIMMYT), ICRAF House, United Nations Avenue, Gigiri, Nairobi, Kenya; USDA, UNITED STATES

## Abstract

We combined the recently developed genotyping by sequencing (GBS) method with joint mapping (also known as nested association mapping) to dissect and understand the genetic architecture controlling stem rust resistance in wheat (*Triticum aestivum*). Ten stem rust resistant wheat varieties were crossed to the susceptible line LMPG-6 to generate F_6_ recombinant inbred lines. The recombinant inbred line populations were phenotyped in Kenya, South Africa, and St. Paul, Minnesota, USA. By joint mapping of the 10 populations, we identified 59 minor and medium-effect QTL (explained phenotypic variance range of 1% – 20%) on 20 chromosomes that contributed towards adult plant resistance to North American *Pgt* races as well as the highly virulent Ug99 race group. Fifteen of the 59 QTL were detected in multiple environments. No epistatic relationship was detected among the QTL. While these numerous small- to medium-effect QTL are shared among the families, the founder parents were found to have different allelic effects for the QTL. Fourteen QTL identified by joint mapping were also detected in single-population mapping. As these QTL were mapped using SNP markers with known locations on the physical chromosomes, the genomic regions identified with QTL could be explored more in depth to discover candidate genes for stem rust resistance. The use of GBS-derived *de novo* SNPs in mapping resistance to stem rust shown in this study could be used as a model to conduct similar marker-trait association studies in other plant species.

## Introduction

Wheat stem rust, caused by the fungus *Puccinia graminis* f. sp. *tritici* (*Pgt*) has been a major constraint in the production of this staple crop since the earliest days of its cultivation. Historical records show that the disease is highly damaging during epidemics and is capable of posing a serious threat to global food security. The pathogen is well known for its ability to travel long distances and evolve-in to new virulent forms [1]. The use of genetic resistance has remained as one of the economic and environmentally effective strategies to control this disease. However, the pathogen has historically proven that it is able to overcome the deployed genes, giving rise to disease epidemics in regions where susceptible cultivars are grown [2, 3]. The recent evolution and spread of the virulent African stem rust race “Ug99” (TTKSK) and its derivative races (commonly known as the ‘Ug99 race group’), is the latest example of this phenomenon. Race TTKSK and 12 other variants within the same lineage have already defeated resistance genes that had been effective for several decades [4–8]. As predicted by wind trajectories, the arrival of these stem rust races in the breadbaskets of the world is quite likely [1]. Also, the *Pgt* races in North America are changing, as documented by detection of a highly virulent race, TTTTF, in 2000 [9]. Therefore, discovery of new sources of resistance and their deployment is an essential process in order to mitigate this constantly evolving pathogen system.

The nature of genetic resistance to stem rust of wheat is mainly qualitative, in the form of major genes derived from hexaploid bread wheat and related species. All-stage resistance (also known as seedling resistance/race specific resistance) has been a significant part of stem rust resistance breeding. The stem rust resistance gene *Sr12* in the University of Minnesota cultivar ‘Thatcher’, for instance, protected majority of the wheat acreage from stem rust epidemics in the northern US wheat growing regions in the 1930’s and 1940’s [3]. Varieties with the gene *Sr31*, bred and distributed by CIMMYT from the mid-1960s, were popular globally until they were defeated by the highly virulent *Pgt* race TTKSK (Ug99) in 1998 [10]. In contrast to all-stage resistance, adult plant resistance (APR) is considered to be effective against a wider array of *Pgt* races, and is assumed to be durable, mainly because of the race nonspecific effectiveness of APR genes [11, 12]. APR is triggered when the plant reaches boot stage and continues to be effective during important plant growth and development phases, particularly at flowering and during grain filling. To date, only five APR genes (*Sr2*, *Sr55*, *Sr56*, *Sr57*, and *Sr58*) have been discovered [13–17]. Several of the APR genes confer minor effects with 5–20% reduction in disease severity [18]. Therefore, pyramiding few APR genes and all-stage resistance genes together could be an effective disease control strategy [18, 19]. It is important to identify and characterize new sources of resistance which can be useful in breeding resistant varieties. Discovery of genes in breeding lines and adapted germplasm would readily facilitate the use of such genes in breeding programs.

Of various strategies implemented to map causative loci for segregating traits, nested association mapping (NAM) is used to map loci in a multi-cross mating design where one common parent is shared among all other ‘founder’ parents. Also known as joint mapping or joint linkage mapping, this strategy uses the strengths of both linkage mapping and linkage disequilibrium mapping to provide higher mapping power and resolution. Briefly, the strategy involves crossing several founder lines to a single common parent to generate segregating progenies in multiple populations. The genetic background is normalized by the virtue of having a common parent, which allows mapping of segregating alleles in different populations with reference to common-parent specific alleles [20, 21]. Therefore, joint mapping helps to minimize problems that may arise due to genetic heterogeneity, different environmental effects, or simply experimental and sampling differences as all populations are connected by a common parent. The NAM design has also been shown to detect QTL with various effect sizes, including rare alleles, because of its higher statistical power [21, 22]. The efficacy of a NAM design in mapping important QTL has been demonstrated in recent studies in maize [23, 24], Arabidopsis [25], and in a few other crop species [22].

Single nucleotide polymorphism (SNP) markers are preferred over other marker systems for genotyping in genomic studies because of their abundance, low cost per data point, and they are amenable to high-throughput technologies. However, genotyping of populations using SNPs from pre-designed assays is known to introduce founder ascertainment bias and is known to result in less accurate and biased results [26, 27]. The genotyping by sequencing (GBS) approach allows for discovery of high quality population-specific SNPs for genomic studies that are free from ascertainment bias. This process uses restriction enzymes for targeted complexity reduction of genomes followed by next-generation sequencing of multiplexed samples and SNP-calling [28, 29]. This approach is also appealing because of the low cost per sample, relatively faster turnaround time, and malleability in terms of sequence manipulation and data mining. This technique has been successfully used in wheat studies to obtain *de novo* genetic maps [30], and in barley to map alleles influencing plant height [31].

In this study, we use a spring wheat NAM panel composed of ten bi-parental recombinant inbred line (RIL) populations to conduct a genome-wide scan for stem rust resistance QTL in a joint analysis of all ten populations. We use GBS markers with known locations on the physical map for the first time to conduct a NAM analysis in a crop species to dissect the relationship between the genetics of a large mapping population and their resistance to African and N. American *Pgt* races. All founder parents used in the crosses have been released as commercial wheat varieties in the past in their target areas. As such, we expect that the resistance loci identified in this study will provide higher value and incentive towards the application of the detected QTL for resistance breeding against stem rust of wheat.

## Materials and Methods

### Plant Material

Nine Kenyan spring wheat cultivars (‘Kenya Fahari’, ‘Gem’, ‘Kenya Kudu’, ‘Kulungu’, ‘Kenya Ngiri’, ‘Kenya Paka’, ‘Pasa’, ‘Kenya Popo’, and ‘Romany’) that were released in the period 1964–1989 were selected for crossing based on the high level of APR exhibited by these lines during screening in the Njoro stem rust nursery in Kenya (Table 1). ‘Ada’, a recent hard red spring wheat variety released by the University of Minnesota [32], also exhibits moderate resistance to the Ug99 race group and a high level of resistance against N. American *Pgt* races (Table 1). These ten lines were crossed to the stem rust susceptible line ‘LMPG-6’ to develop RIL populations (F_6_ or more inbred) via the single seed descent method at the University of Minnesota (S1 Fig). The number of inbred lines in the ten populations ranged from 55 to 110 (Table 1). Hereafter, the lines Kenya Fahari, Kenya Kudu, Kenya Ngiri, Kenya Paka, and Kenya Popo are referred to as Fahari, Kudu, Ngiri, Paka, and Popo, respectively.

**Table 1:** Origin, pedigree, and stem rust reaction of parent lines used to develop the NAM population.

Parent	Origin^a^	Pedigree^a^	TTKSK^b^	QFCSC^b^	MCCFC^b^	TPMKC^b^	TTTTF^b^	Field reaction^c^		No. of RILs
								USA	Africa	
Ada	USA (2007)	SBY189H/‘2375’	3+	;1-	2-	2	0;/;1	22.3	11.3	71
Fahari	Kenya (1977)	TOBARI-66/3/SRPC-527-67//CI-8154/2*FROCOR	33+	0	;	0	0	11.7	3.7	90
Gem	Kenya (1964)	BT908/FRONTANA//CAJEME 54	3-	0	0	0	3–1	10.2	1.0	97
Kudu	Kenya (1966)	KENYA-131/KENYA-184-P	3+	0	;2-	0;/2-	31	19.8	3.7	80
Kulungu	Kenya (1982)	ON/TR207/3/CNO//SN64/4/KTM	33+	0	0	0	0	22.5	0.3	59
Ngiri	Kenya (1979)	SANTACATALINA/3/MANITOU/4/2*TOBARI-66	33+	0	0	0	0	10.2	2.3	52
Paka	Kenya (1975)	WISCONSIN-245/II-50-17//CI-8154/2*TOBARI-66	3+	0	0;	0	0	16.5	3.7	104
Pasa	Kenya (1989)	BUCK BUCK/CHAT	3+	0;	0	2-;	2-	8.6	5.3	93
Popo	Kenya (1982)	KLEIN-ATLAS/TOBARI-66//CENTRIFEN/3/BLUEBIRD/4/KENYA-FAHARI	3+	0	0	0;	0	5.0	2.3	97
Romany	Kenya (1966)	COLOTANA 261-51/YAKTANA 54A	3+	0	0	0	0	11.1	5.3	109
LMPG-6	Canada (1990)	LITTLE-CLUB//PRELUDE*8/MARQUIS/3/GABO	4	4	4	4	4	64.9	25.0	-

^a^ Information obtained from Njau et al. [90], Macharia [66], Anderson et al. [32], and Knott [91]. Year (in parenthesis) indicates the year the line was released or published.

^b^ Seedling screening of the parent lines with African stem rust race Ug99 (TTKSK, isolate ‘04KEN156/04’), and N. American stem rust races QFCSC (isolate ‘06ND76C’), MCCFC (isolate ‘59KS19’), TPMKC (isolate ‘74MN1409’), and TTTTF (isolate ‘01MN84A-1-2’). Infection types are scored on a 0 to 4 scale where 3 and 4 are considered susceptible [92].

^c^ For USA environment, the rust response of parent lines were averaged from StP12 and StP13 environments. For Africa environment, only Ken13 data is shown. Mean severity reactions (%) are shown for both sites.

### Stem Rust Evaluation

The ten RIL populations, comprised of 852 lines, were evaluated for APR to stem rust in four environments: St. Paul, MN, USA during May–August 2012 & 2013 (referred as StP12 and StP13 in the text); South Africa during October 2012–January 2013 (referred as SA12); and Njoro, Kenya during June–October 2013 (referred as Ken13). The stem rust data collected on all populations in all four environments are available in S1 File.

In the Njoro nursery, lines were planted in an augmented design with the susceptible check ‘Red Bobs’. Each line was sown in double rows 70 cm long and 20 cm apart. On each side and in the middle of the plots, a twin-row of susceptible spreader wheat cultivar ‘Cacuke’ was sown. The field was also surrounded by a border of several spreader rows comprised of susceptible wheat varieties that were artificially inoculated using a bulk inoculum of *Pgt* urediniospores collected at the Njoro field site. The wheat stem rust differential lines with known stem rust resistance genes indicated that the predominant, if not only, race present in the nursery was TTKST (avirulence/virulence formula on the wheat stem rust differential panel: *Sr36*, *SrTmp*/*Sr5*, *Sr6*, *Sr7b*, *Sr8a*, *Sr9a*, *Sr9b*, *Sr9d*, *Sr9e*, *Sr9g*, *Sr10*, *Sr11*, *Sr17*, *Sr21*, *Sr24*, *Sr30*, *Sr31*, *Sr38*, *SrMcN*) [33].

In the St. Paul 2012 environment, lines were planted in 2 m long single rows with 20 cm between the rows. The populations were planted in an augmented design with 4 check varieties ‘Oklee’ [34], ‘Thatcher’ [35], ‘Tom’ [36], and ‘Verde’ [37] planted after every 30 entries. In the St. Paul 2013 environment, lines were planted in hill-plots with 20 cm distance between the hills. The same checks were planted in the same manner as in the 2012 season. In both St. Paul environments, the lines were surrounded by a mixture of susceptible lines ‘Morocco’, ‘Max’, and ‘Little Club’ planted perpendicular to the lines on all sides. To initiate disease, spreader rows were syringe-injected at the jointing stage with a mixture of N. American stem rust races MCCFC (isolate 59KS19), QFCSC (isolate 03ND76C), QTHJC (isolate 75ND717C), RCRSC (isolate 77ND82A), RKQQC (isolate 99KS76A), and TPMKC (isolate 74MN1409). The spreader rows were sprayed with a bulked mixture of *Pgt* races suspended in a light mineral oil suspension using an Ulva+ sprayer (Micron Sprayers Ltd., Bromyard, UK) after heading stage.

In South Africa, populations were planted in Cedara in KwaZulu-Natal Province in an augmented design with the check lines ‘Morocco’ and ‘Kariega’ planted every 50 entries. Seeds for each line were planted in hill plots with 30 cm between plots in a row and 60 cm between rows. To initiate the disease, spreader rows containing ‘Morocco’ and ‘McNair’ were inoculated with a mixture of the *Pgt* races TTKSF and PTKST of the Ug99 race group using an ultra-low volume sprayer twice in the season—once during the booting stage, and again at flowering stage.

Field reaction of the RILs to stem rust were recorded as disease severity on the 0 to 100 modified Cobb scale [38], and infection response, based on the size of pustules and amount of chlorosis and necrosis visible on the stem [39]. Disease phenotyping of the population segregating for resistance was carried out after the susceptible check varieties in each trial had attained maximum severity. Following Stubbs et al. [40], the severity response value was multiplied by the infection response to obtain coefficient of infection (CI) values. The number of days to heading (in StP12 and StP13) and plant growth stage (in Ken13) were also recorded. The number of days to heading was measured as the day after planting when half the spikes in the plot fully emerged above the flag leaf. Plant growth stages were determined mainly by assessing grain development stages such as watery, milky, soft dough, and hard dough; and also for stages of booting and flowering, as explained by Zadoks et al. [41]. Additionally, all 11 parental lines were inoculated with race TTKSK (isolate 04KEN156/04) at the seedling stage to postulate the presence/absence of major genes providing resistance to this race. Disease inoculation and phenotyping procedures were carried out as described by Rouse et al. [42].

### Statistical Analysis

**Fig 1:**
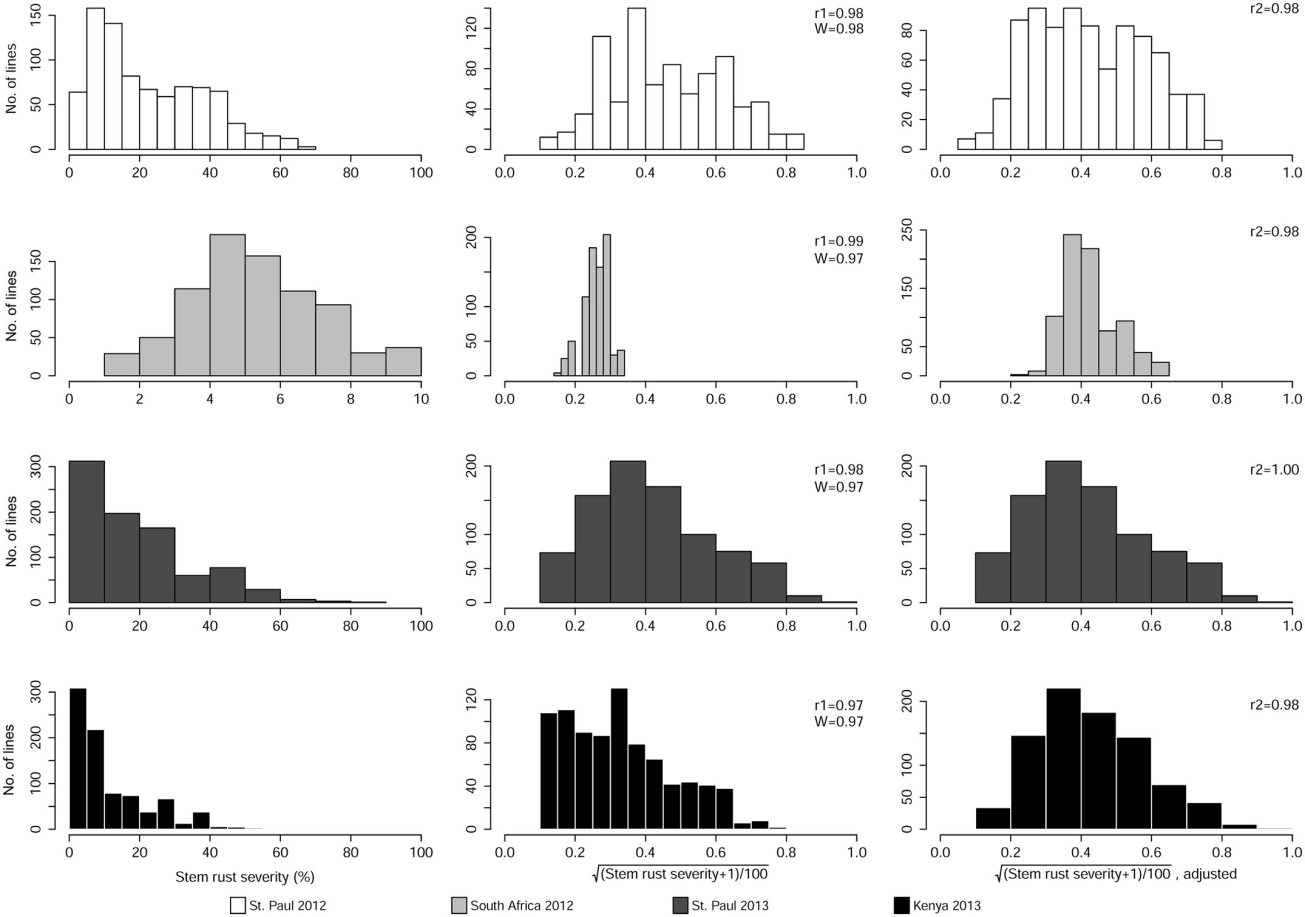
Distributions for disease coefficient of infection (CI) and their respective transformed datasets for stem rust in each of the four environments. The Pearson’s correlations are represented as follows: “r1” between the coefficient of infection (CI) values and square root transformed data; and “r2” between the square root transformed data and data adjusted for trial differences. “W” represents the Shapiro-Wilk test statistic between the coefficient of infection (CI) values and square root transformed data.

To improve normality of the phenotypic data, arcsine-square root, log, and square root transformation methods were tested. The CI values were passed through the formulae [arcsine (√ 1-(CI+1)/100)], [log(CI+1)], and [(√ (CI+1)/100)] for arcsine-square root, log, and square root transformation, respectively. Each transformed dataset was tested for improved normality using the Shapiro-Wilk normality test. While the test confirmed that the transformed data improved in normality (Fig 1), significant departures from normality were still observed (S1 File). Based on the normality test, the square root transformed datasets were used in the next steps. Using genotypes as fixed effects, mixed linear models were then fitted to assess family and line-within-family effects, and also the effects due to differences in heading date (St. Paul data) and plant growth stages (Kenya data). Factors explaining significant amounts of variation were retained in the model and the effect estimates for each line was done using *lme4* package (version 1.0–6) in R 3.0.3 (R Development Core Team 2013, http://www.r-project.org/).

The phenotypic scale used in South Africa was different than in other locations as the disease was evaluated on a scale of 0 to 10 with scores: 0–2 (highly resistant), 3 (resistant), 4–5 (moderate resistant), 6–7 (moderate susceptible), 8 (susceptible), and 9–10 (highly susceptible) [43]. Therefore, all data sets (fixed effects estimated using *lme4* for each environment) were fitted into a mixed model in SAS 9.1 (SAS Institute Inc, Cary, NC, USA) with environments as fixed effects and lines as random effects. Thus estimated best linear unbiased predictors (BLUPs) were used in QTL mapping. Heritability across all environments was calculated on an entry mean basis [44].

### Genotyping and SNP Discovery

All lines used in the study were genotyped using the genotyping by sequencing (GBS) approach [28]. DNA was extracted from ground leaf tissue of parents and F_6:7_ RILs using the BioSprint 96 DNA Plant Kit (QIAGEN, Valencia, CA). Extracted DNA was quantified using the picogreen assay and diluted to 20 ng/μl. The restriction enzymes *Pst*I and *Msp*I were used to generate double-digested complexity-reduced libraries following Poland et al. [29] with few modifications: 1) each sample was ligated to two unique barcodes to minimize sequencing bias of reads with certain barcodes; and 2) the concentrations of both barcode and common adapters were increased to 0.1 μM and 50 μM, respectively, in an attempt to capture most of the digested DNA fragments. Ten 96-plex libraries were generated, with each parent repeated at least six times to obtain higher read coverage of parental alleles. Each library was sequenced in one lane of Illumina HiSeq 2000, generating 100 bp single-end sequences. Sequences of all 11 parents and RILs of all 10 populations have been uploaded to NCBI’s sequence read archive (SRA: SRP057693; BioProject: PRJNA281776).

For each population, sequences of each RIL and the two parents were aligned to the Chinese Spring genome assembly [45] ordered into chromosomal pseudomolecules using population sequencing (POPSEQ) data generated by Chapman et al. [46]. Sequence alignment was carried out using the *aln* method in ‘bwa’ using default parameters. The software ‘Samtools’ was used to process the aligned sequences (*view* method) followed by SNP calling (*mpileup* method). SNPs were accepted if the alignment read depth was ≥ 3 and the minimum mapping quality was ≥ 25. Heterozygote calls were converted to missing allele calls to allow for the imputation algorithm to discern either parental genotype. The workflow of genotyping, map construction, data analysis, and genetic mapping in this study is presented in S1 Fig.

### Map Construction and Joint QTL Mapping

Genetic maps for individual populations as well as the combined panel were constructed using IciMapping 4.1 [47] with a minimum logarithm of odds (LOD) value of 5.0. Genetic distances between the markers were calculated based on the Kosambi mapping function [48]. For each population, an initial map was constructed comprising only of SNP markers with proportion of missing alleles at ≤ 20% and minor allele frequency of ≥ 30%. To this, markers with missing data of up to 50% were added and the linkage groups were reordered. To construct a joint map combining all populations, SNP markers present in at least two RIL populations were used. Construction of joint map was carried out as explained above. Imputation of the missing alleles was not carried out prior to map construction and the native imputation algorithm within IciMapping was implemented during QTL mapping to reconstruct the missing haplotypes. This algorithm assigns parental genotypes to the missing markers by considering linkage relationship between markers based on their map order on each chromosome (or linkage group) within each family [49]. Genotypic data on the joint panel—both non-imputed and imputed—has been provided as supplemental information (S2–S7 Files). A joint QTL mapping across all populations was done using the joint inclusive composite interval mapping (JICIM) method in IciMapping 4.1. JICIM, an efficient and specialty method for joint QTL mapping for the NAM design, offers higher mapping power and can therefore detect small-effect QTL better [23, 50]. Within JICIM, presence of QTL along the chromosomes was scanned at an interval of 5 cM and a QTL was declared significant if the threshold was greater than the 1,000 permutation of the trait data by resampling method [51], at type I error α = 0.05. The thresholds for StP12, StP13, SA12, and Ken13 were estimated at 4.3, 4.5, 5.8, and 4.8, respectively. Epistatic effects among the detected QTL were estimated using the joint connected model in MCQTL 5.2.6 [52] by providing the QTL information, yet a lower LOD threshold of 3.0 was chosen. QTL mapping in each population was also carried out separately using the inclusive composite interval mapping (ICIM) approach in IciMapping 4.1 using LOD threshold of 3.0. For each detected QTL, the percent of phenotypic variance explained (R^2^) and allelic effects were also estimated.

## Results and Discussion

### Genotyping

We generated ten 96-plex GBS libraries representing 852 RILs from ten biparental populations. The sequencing of each library on one lane of Illumina HiSeq 2000 generated a total of 1.5 billion 100 bp reads, with 154 million reads on average per lane. On average, 90% of the generated bases passed the Q30 filter with a median Q-score of 34.98. The reads were then filtered for having intact barcode sequences and a complete *Pst*I overhang, which led to 73% of total reads assigned to each individual. This is comparable to recent GBS studies in wheat [30] and barley [31] that follow the same library construction and sequencing protocols. This resulted in a read distribution per individual from 175,443 to 31,381,071 with a median of 1,225,681 reads per RIL. Each parent line was sequenced at least six times to obtain sequences with higher read depth for confidence in SNP-calling and imputation. As a result, a total of 158,182,462 reads were obtained for the 11 parents used in the study, with a median value of 13,348,322 reads, and ranging from 7,951,649 (Kudu) to 31,381,071 of the common parent LMPG-6 which was replicated 10 times (S1 File).

### SNP Discovery, Linkage Mapping, and Segregation Distortion

After calling SNPs within each population using the Samtools program, SNPs were renamed in the format ‘*Chr_position*’ to reflect the base position on the reference chromosome. SNPs within each population were filtered to obtain SNPs with: a) nucleotide base as an allele for both parents and b) two parents were polymorphic for the SNP call. The amount of missing data in the resulting files ranged from 0% to 100%, with SNP counts ranging from 29,651 (LMPG-6/Kudu) to 41,418 (LMPG-6/Popo) (S1 File). Common SNPs between at least two populations, identified as the ones with the same SNP name as described above, were selected to create the joint linkage map. This led to identification of 13,413 SNPs that were used for construction of linkage maps. Of these, 11,221 SNPs were assigned to 21 linkage groups with all 21 wheat chromosome represented. The number of markers per linkage group ranged from 138 (Chromosome 3D) to 1,218 (Chromosome 3B). The total genetic distance covered by these groups was 1,920 cM, with one SNP marker placed at every 0.17 cM on average. Most SNPs were assigned to the B genome with 5,259 SNPs, followed by the A genome (3,871) and the D genome (2,091) (S1 File). Though the D genome is usually considered to be the least polymorphic of all wheat genomes [53, 54], our approach of reference-based SNP-calling may have helped to greatly increase the marker coverage (1 marker every 0.18 cM, on average) for this genome. It is also worth noting that the GBS approach has been shown to provide a better coverage of the D genome by earlier studies [29, 55].

**Fig 2:**
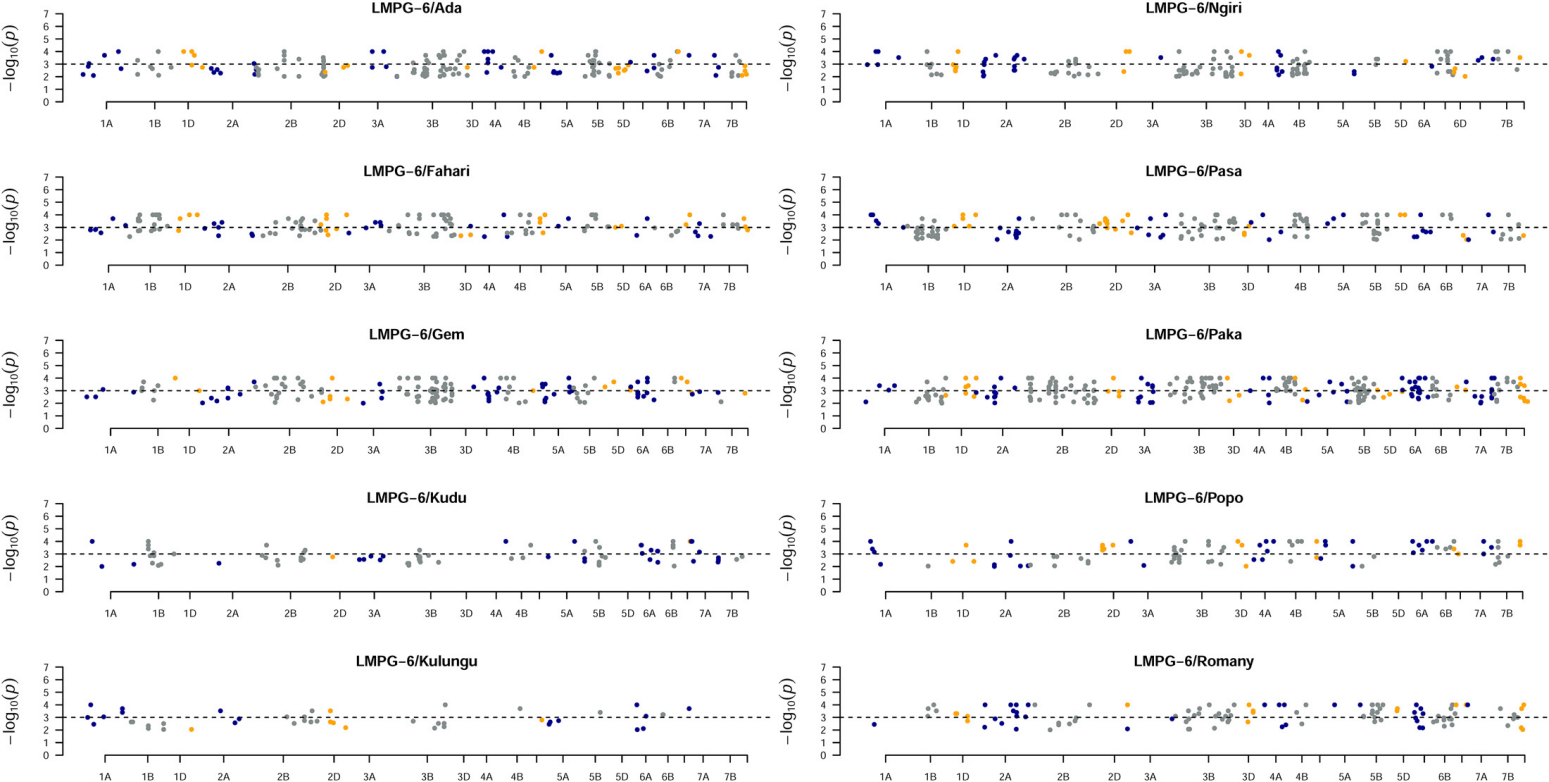
Segregation distortion of loci across each RIL mapping population. Chromosome names and the—log(*p*) value for SNP markers in respective chromosomes for each population is shown.

As we used markers present in two or more populations to create the combined map, an accurate estimation of segregation distortion was affected by a large amount of missing alleles. The same held true for individual population maps where markers with up to 50% missing data were retained. While imputation of the alleles partly aided in giving better distortion ratio, it was not able to solve the problem completely. Therefore, we report here the segregation distortions in each family based on the combined map when the missing data per family was ≤ 50%. While this still does not present a clear picture, all populations can be compared by the virtue of the same map. Several markers in most chromosomes in each RIL population deviated significantly (*p* <0.001), yet no strong distorted pattern was visible at any particular locus on any chromosome in any population (Fig 2). The population LMPG-6/Ada had the lowest amount of segregation distortion at 32.0%, whereas LMPG-6/Romany had the highest (58.5%). The average amount of distortion across all RIL populations was 44.7%. These numbers are slightly higher in comparison to the numbers reported a recently published study that used GBS to create a consensus map (albeit in different populations than ours with much less missing data) and study several disease traits in wheat [56]. We believe that using markers with less or no missing data can more accurately illustrate the deviations from the expected segregation ratio of 1:1 distortions, especially for markers at higher significance levels (*p* <<0.001).

**Fig 3:**
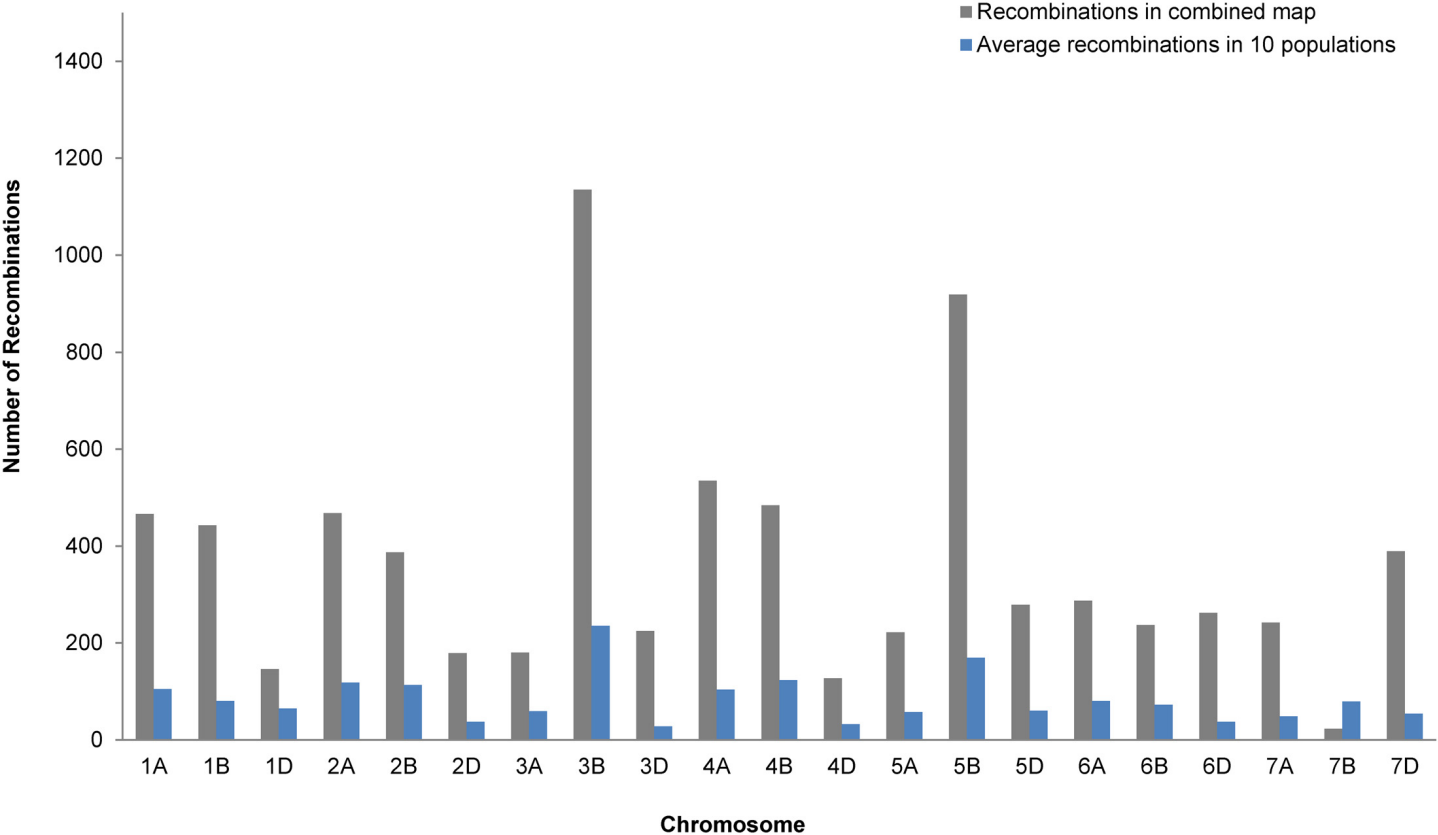
Number of recombination events per chromosome in the joint map (gray bar) relative to the average number of recombinations per chromosome in all 10 populations.

An advantage of using a joint linkage map that combines several populations is that more recombination events are obtained per chromosome. This was evident in our joint panel as higher recombination frequencies were observed on each chromosome, relative to that in the individual populations (Fig 3). While an increased capture of recombination events leads to higher resolution in QTL mapping, it can also potentially unlock hidden genomic regions, thereby allowing for detection of otherwise unmapped QTL.

### Population Characteristics and Stem Rust Reaction

Population structure among the populations was determined by singular value decomposition of the genotype data using the *princomp* package in R 3.0.3 (R Development Core Team 2013, http://www.r-project.org/). No significant structuring among the lines was observed (S2 Fig), which is expected of the NAM population design [21]. The lack of structure among the lines can also be credited to the parents, which are diverse from each other (S3 Fig). The lack of population structure among the populations despite having diverse founder lines is an attractive aspect of the NAM design. As different founder lines are crossed to a common parent, shuffling of the parental genomes in the progeny normalizes allelic differences by virtue of the common parent. Therefore, population stratification is effectively controlled in the NAM design, which minimizes spurious associations that could arise from population structure. Because of the lack of structure, relationship matrices were not used as cofactors during joint mapping.

All ten RIL populations and the parent lines were phenotyped over two years in four environments where disease epidemics were artificially established by inoculating spreader lines with local *Pgt* races. Screening for resistance to N. American and African *Pgt* races showed that all male parents used in the cross were resistant to moderately resistant (Table 1). Some susceptible pustules were observed on the stems of ‘Ada’ in the Kenya 2013 nursery, albeit at low severity (average of 11%). The female parent ‘LMPG-6’ succumbed to high disease severity in all four environments and exhibited susceptible infection responses. Phenotyping of all populations was initiated once disease severity on ‘LMPG-6’ was at its maximum at each environment. The frequency of resistant lines was higher in the Kenya 2013 environment than any other (Fig 1, S4 Fig), most likely due to the unusually cooler day and night temperatures at this site during the 2013 main season, which led to slower onset of disease than usual. Adjustment of phenotypic data was able to resolve this, as trait means among all environments were similar post-adjustment. The broad sense heritability across four environments was estimated at 0.55.

Seedling screening of the parent lines with the race TTKSK (Ug99) showed that all parents were susceptible to this race (Table 1). Seedling susceptibility of the parents to TTKSK indicated that the lines lacked seedling resistance genes effective against this race. The parent lines were screened for the presence of *Sr2* using available DNA markers [57], and also for the presence of another APR gene, *Sr57*, using marker *csLV34* of the linked leaf rust APR gene *Lr34* [58]. Results indicated that four of the ten founder lines—‘Gem’, ‘Kudu’, ‘Paka’, and ‘Romany’—could contain *Sr2*, whereas none of the parents appeared to have *Sr57*. Marker screening of other APR genes, namely *Sr55*, *Sr56*, and *Sr58*, was not possible due to the unavailability of diagnostic markers.

### Stem Rust QTL Mapping

**Fig 4:**
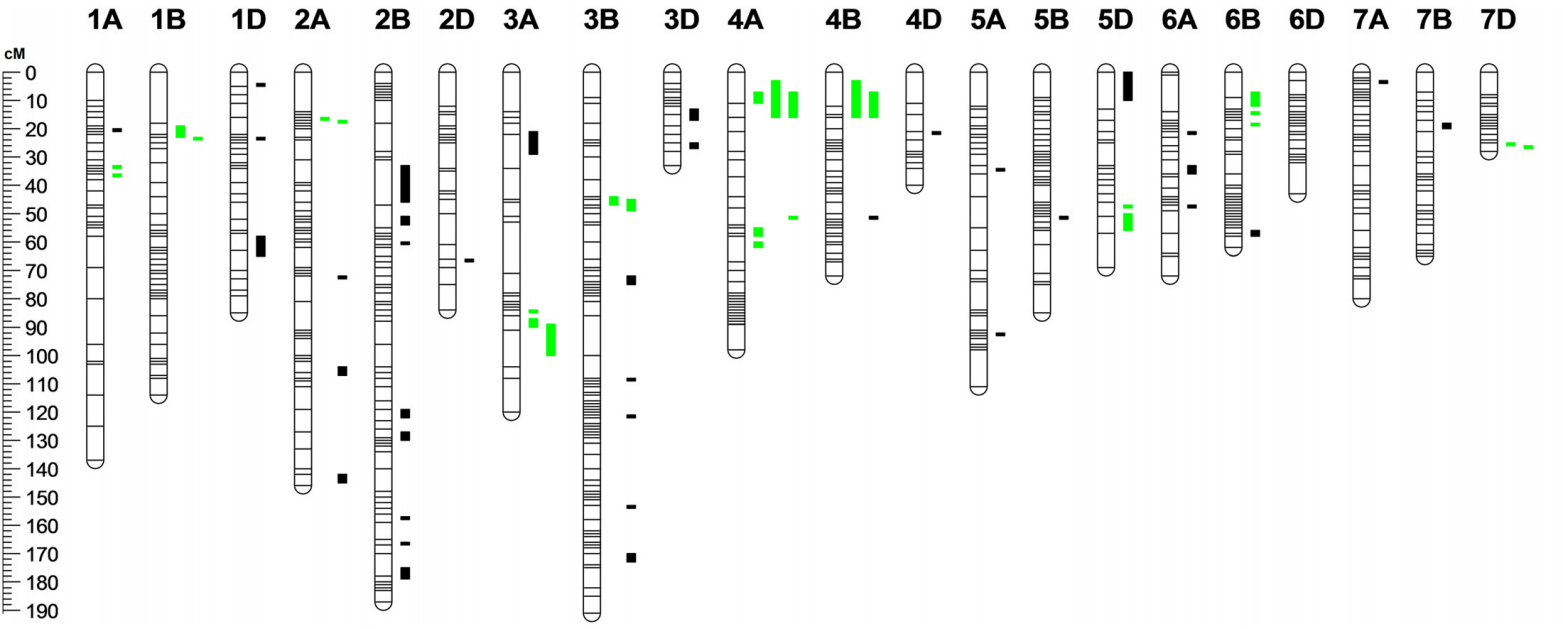
Chromosomes with APR QTL to stem rust detected by the joint inclusive composite interval mapping (JICIM) method. Multiple QTL in green color on a given chromosome are hypothesized to be a single QTL.

The joint inclusive composite interval mapping (JICIM) approach discovered 59 QTL on 20 of 21 wheat chromosomes (Fig 4, S1 File). Though, the ‘effective’ number of QTL could be 48 if adjacent QTL (highlighted in green in Fig 4) on a few chromosomes are considered as a single QTL because of their proximity to each other. Most QTL (26) were detected on B genome chromosomes. The number of QTL detected in A and D genomes were 21 (on all A genome chromosomes) and 12 (on all D genome chromosomes except 6D), respectively. The detection of fewer number of QTL in D genome was quite interesting, and may be attributed to a lower coverage of this genome relative to the other two genomes. Despite the D genome having a generous number of markers mapped to its chromosomes, the genomic distance covered by these markers was only 50% relative to that of the A and B genomes. Fifteen of the 59 QTL were detected in more than one environment: *QSr*.*umn-1B*.*2* and *QSr*.*umn-5A*.*2* were common between Ken13 and StP13 environments; *QSr*.*umn-2A*.*4*, *QSr*.*umn-2B*.*3*, *QSr*.*umn-2B*.*8*, *QSr*.*umn-3B*.*1*, *QSr*.*umn-3B*.*3*, *QSr*.*umn-3B*.*4*, and *QSr*.*umn-5B* between StP12 and StP13; *QSr*.*umn-2B*.*2* between Ken13 and SA12; *QSr*.*umn-3B*.*5* between SA12 and StP12; *QSr*.*umn-6B*.*2* between StP12 and Ken13. The QTL *QSr*.*umn-5D*.*1*, *QSr*.*umn-6B*.*4*, and *QSr*.*umn-7B* were common in three environments: StP12, StP13, and Ken13; No QTL was common among all four environments.

**Fig 5:**
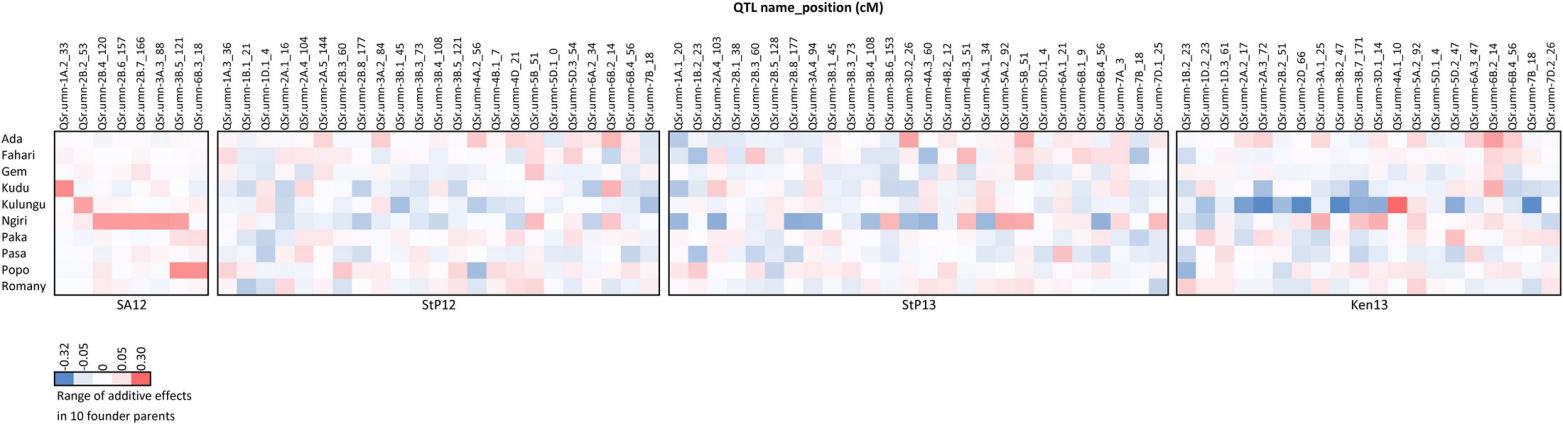
Heat map of additive effect estimates of alleles contributed by the 10 founder lines at the QTL for resistance to *Pgt* races. QTL (columns) are named according to McIntosh et al. [89] with their chromosomal positions after the underscore (_) symbol. The allelic effect estimates for each founder allele (rows) are color coded by increments in the allelic effect estimate (legend). Each block represents the environments where the QTL were detected, as labeled.

The detected QTL explained a wide range of phenotypic variation (R^2^), with the lowest R^2^ of 1.4% and the highest of 20.4%. These values indicate the presence of small to medium-effect QTL contributing towards stem rust resistance. However, small population sizes can inflate phenotypic estimation variation [59]. As some RIL populations in our study are small (smallest population with n = 52), the R^2^ values as well as the additive effect estimates could have greater error vs. populations of larger size. The additive effect estimates for each QTL were of small magnitude (S1 File), a function of the scale of transformed data. For each QTL, the parental alleles contributed different effects towards stem rust resistance, as exhibited by the distribution of total additive phenotypic effects of each parent (Fig 5). Overall, each parent contributed towards resistance in each environment. The positive values associated with the parent lines for specific SNP markers in certain environments do not imply that the parents are not important sources of resistance. As the effects of all parental alleles are considered simultaneously during joint mapping, variations in disease response in each family, contributed by each parent, are expected to occur. Moreover, the choice of a parent for introgression of alleles for APR is also a function of several important factors defining the target environment such as frequency, and types of *Pgt* races, and disease pressure. While the contribution to disease resistance from alleles with low additive values might be difficult to visually observe in the field, APR genes acting in an additive manner should elevate the resistance, thereby assisting in phenotypic selection. Likewise, combining QTL that were common in multiple environments is also likely to lead towards significant reduction in disease damage.

No epistasis was detected among QTL within the populations, neither in a joint setting with all populations combined. We did however observe few non-QTL markers on a few chromosomes with LOD scores in the range of 2.5–2.8. Therefore, the main reason for no significant epistasis is likely due to the stringent threshold (LOD value ≥ 3.0) required for declaration of such interaction. Recent studies have mixed results for detection of epistatic interactions between the mapped QTL as some studies report significant levels of interaction [60–63] whereas other studies do not [64–66]. Taking together the larger number of detected QTL and the small magnitude of the marker effects, the nature of resistance to stem rust in these ten populations could be assumed to be polygenic, with several loci acting in additive fashion. A point to consider is that the lack of epistasis should not deter a breeder from adoption of a QTL as long as it contributes to elevated APR levels.

### Comparison of Joint Mapping to Single-population QTL Mapping

The ICIM approach of QTL detection conducted on each RIL population detected a total of 59 QTL on 20 chromosomes (no QTL detected on 3D) (S1 File). The populations LMPG-6/Gem and LMPG-6/Romany had the most QTL (13), and the population LMPG-6/Ngiri had the least (1); no QTL was observed in LMPG-6/Popo. There were no common QTL among all four environments in any individual RIL population, although four populations shared at least one QTL in three of the four environments. This difference is likely due to the presence of different rust races in the four environments as well as other environmental factors, as evident by the observed genotype by environment (GxE) interactions (S5 Fig).

We were mainly interested in knowing if the QTL detected during joint mapping could also be detected by conducting QTL mapping of each population separately. While only three QTL that were detected by JICIM were detected by ICIM based on flanking markers, a closer look at the QTL positions revealed that as many as 14 QTL on 11 chromosomes could be common between the two methods (Table 2). Nine of these 14 QTL are located on the same position, albeit QTL mapping reported different flanking markers based on the difference in the confidence interval; remaining 4 are within a distance of ≤ 3 cM. Six of the 14 QTL were detected in the same environments between the two methods, and eight were detected in different environments. The difference of the environments where the QTL were detected suggests that these loci might be involved in providing resistance that is specific to each disease environment, and perhaps to specific *Pgt* races. It could also indicate differences in environmental conditions, inoculum load, and difference in genetic backgrounds (e.g. different alleles). S1 Text expounds on this comparison.

**Table 2:** Common quantitative trait loci (QTL) detected between the joint inclusive composite interval mapping (JICIM) in nested association mapping of 10 RIL populations, and inclusive composite interval mapping (ICIM) methods in individual populations for stem rust adult plant resistance in four environments.

Chr^a^	Pos (cM)^b^	Population^c^	Env	Left Marker	Right Marker	LOD^d^	R^2^ (%)^e^	Add^f^
1A	**33**	**Joint**	**SA12**	**1A_53256976**	**1A_214244842**	**10.26**	**3.05**	
	*35*	*Kulungu*	*Ken13*	*1A_64961094*	*1A_101708352*	*4*.*12*	*1*.*84*	-*0*.*15*
	*36*	*Fahari*	*SA12*	*1A_184159449*	*1A_50973716*	*15*.*36*	*38*.*71*	*0*.*22*
	**36**	**Joint**	**StP12**	**1A_168260073**	**1A_50914673**	**5.86**	**4.22**	
	*37*	*Romany*	*SA12*	*1A_39856840*	*1A_235277072*	*3*.*22*	*2*.*20*	*0*.*09*
1D	*20*	*Gem*	*StP12*	*1D_96967944*	*1D_11687876*	*4*.*01*	*1*.*26*	*0*.*15*
	**23**	**Joint**	**Ken13**	**1D_95968008**	**1D_62309262**	**5.01**	**8.16**	
2A	**16**	**Joint**	**StP12**	**2A_13651965**	**2A_195408664**	**4.49**	**8.78**	
	*17*	*Romany*	*SA12*	*2A_11044893*	*2A_152187592*	*3*.*06*	*1*.*31*	-*0*.*04*
	**17**	**Joint**	**Ken13**	**2A_118236938**	**2A_152187592**	**6.68**	**10.63**	
	*20*	*Romany*	*SA12*	*2A_85264302*	*2A_96999682*	*13*.*96*	*6*.*54*	-*0*.*16*
	**144**	**Joint**	**StP12**	**2A_254280385**	**2A_226128547**	**5.31**	**2.40**	
	*145*	*Ada*	*StP12*	*2A_254280385*	*2A_226128547*	*3*.*52*	*7*.*86*	*0*.*05*
2B	**53**	**Joint**	**SA12**	**2B_295548243**	**2B_4526688**	**18.27**	**1.52**	
	*55*	*Romany*	*SA12*	*2B_14347146*	*2B_326925659*	*3*.*57*	*2*.*21*	*0*.*07*
	*60*	*Ada*	*StP13*	*2B_328392028*	*2B_344339504*	*3*.*00*	*3*.*15*	-*0*.*14*
	**60**	**Joint**	**StP13**	**2B_309579404**	**2B_313124463**	**6.62**	**6.73**	
	**60**	**Joint**	**StP12**	**2B_309579404**	**2B_313124463**	**4.68**	**6.91**	
3B	*108*	*Pasa*	*StP13*	*3B_647995685*	*3B_584832142*	*9*.*82*	*10*.*93*	-*0*.*05*
	**108**	**Joint**	**StP12**	**3B_673326253**	**3B_82601403**	**4.90**	**3.16**	
	**108**	**Joint**	**StP13**	**3B_673326253**	**3B_82601403**	**6.89**	**5.00**	
	*109*	*Pasa*	*StP12*	*3B_647995685*	*3B_584832142*	*7*.*12*	*9*.*91*	-*0*.*05*
	*121*	*Gem*	*StP13*	*3B_496875398*	*3B_554937272*	*5*.*70*	*19*.*89*	-*0*.*07*
	**121**	**Joint**	**SA12**	**3B_538190692**	**3B_554937272**	**13.40**	**4.43**	
	**121**	**Joint**	**StP12**	**3B_538190692**	**3B_554937272**	**5.75**	**6.24**	
	*124*	*Kudu*	*StP12*	*3B_591992271*	*3B_909961*	*3*.*65*	*17*.*15*	-*0*.*12*
	*124*	*Kudu*	*StP13*	*3B_591992271*	*3B_909961*	*4*.*21*	*29*.*22*	-*0*.*09*
	*124*	*Kudu*	*Ken13*	*3B_591992271*	*3B_909961*	*3*.*10*	*10*.*93*	-*0*.*12*
4B	*51*	*Romany*	*StP13*	*4B_299160842*	*4B_210047150*	*3*.*57*	*1*.*06*	*0*.*14*
	**51**	**Joint**	**StP13**	**4B_282191767**	**4B_203809435**	**5.89**	**8.27**	
4D	*21*	*Kulungu*	*Ken13*	*4D_50470445*	*4D_59436083*	*3*.*51*	*1*.*93*	-*0*.*16*
	**21**	**Joint**	**StP12**	**4D_61244682**	**4D_58434790**	**4.62**	**7.73**	
5B	**51**	**Joint**	**StP13**	**5B_258358007**	**5B_233185212**	**7.69**	**8.42**	
	*53*	*Gem*	*StP12*	*5B_257695597*	*5B_155513783*	*4*.*94*	*1*.*28*	-*0*.*14*
5D	**47**	**Joint**	**Ken13**	**5D_145713577**	**5D_101495576**	**6.28**	**13.20**	
	*48*	*Kulungu*	*Ken13*	*5D_27462012*	*5D_91682679*	*3*.*33*	*1*.*88*	-*0*.*16*
6A	*21*	*Pasa*	*SA12*	*6A_41412787*	*6A_49007339*	*11*.*68*	*6*.*71*	-*0*.*02*
	**21**	**Joint**	**StP13**	**6A_12874936**	**6A_16883183**	**4.87**	**4.33**	
7A	*3*	*Gem*	*StP12*	*7A_92272971*	*7A_134846357*	*3*.*94*	*1*.*05*	-*0*.*15*
	**3**	**Joint**	**StP13**	**7A_5990339**	**7A_2150377**	**5.96**	**3.15**	

^a^ Chromosome location of the QTL.

^b^ Position (centiMorgan) of the detected QTL peak in Chromosome ‘Chr’. Positions are sorted in ascending order.

^c^ Result of JICIM is shown in bold; result of ICIM is shown in italics.

^d^ Logarithm of odds scores for the QTL detected at position ‘Pos’, based on joint mapping.

^e^ Percentage of phenotypic variation explained by the observed QTL, based on joint mapping.

^f^ Additive effect for JICIM is not shown as JICIM reports additive effect for each parent individually; see S1 File for more information.

### Comparison of Joint Mapping Results to Previously Reported Genes and QTL Conferring Resistance to Pgt

Kenyan lines are known to be the sources of many *Sr* genes discovered to date [3]. Examples include *Sr6*, which is quite common in Kenyan lines [67], and *Sr9b*, which was first observed in Kenyan lines [68]. The line ‘Frontana’, present in the pedigree of the founder parent ‘Gem’, is known as one of the sources of *Sr9b* [3]. Hence, the NAM panel presented here may possess these *Sr* genes, in addition to previously unidentified genes. In an effort to explore the relationship among the QTL detected in our study and previously reported stem rust genes and QTL, we BLASTn searched sequences containing the SNP markers associated with QTL in our study against the Infinium iSelect 9K [69], 90K [70] SNP sequences, and the sequences of GBS-SNPs from the consensus map comprising of GBS-SNP and SSR markers developed by Saintenac et al. [30], in an attempt to determine the position of QTL detected in our study relative to those reported in peer-reviewed studies. However, this yielded in zero high-quality matches among the sequences; although alignments with high number of mismatches and alignment lengths of less than 50% were observed. Additionally, the markers associated with QTL were searched against the putative wheat gene models (v2.2) made available on Ensembl; no matches were found. Though the SNP markers flank a given QTL, they (and the sequences housing the SNP) might not necessarily be of high importance as they are in essence flanking a possible genic region on the chromosome. Therefore, further investigation of the flanked chromosomal regions will likely lead to gene discoveries as well as a better understanding of the nature of disease resistance. While we are unable to draw direct comparisons with previously published QTL mapping studies, our methodology of reference based SNPs used in mapping, thus obtained mapping results, as well as comparative mapping of GBS SNPs with the annotated reference wheat genome in near future could be important steps in the direction of identifying regions with potential candidate genes.

Three QTL were detected on 1A in two environments: StP12 and StP13; two QTL on 1B in three environments: Ken13, StP12, and StP13, of which *QSr*.*umn-1B*.*2* was common between StP13 and Ken13; and three QTL on 1D in two environments: Ken13 and StP12. Previous studies that have reported QTL associated with APR to stem rust include: Rouse et al. [63] in Thatcher wheat, Bhavani et al. [64] in the CIMMYT bi-parental population PBW343/Kingbird, Yu et al. [60] in CIMMYT’s winter wheat breeding germplasm, Pozniak et al. [71] in durum wheat (*Triticum durum* Desf.) association mapping panel, and Singh et al. [62] in the durum wheat population Sachem/Strongfield. The TTKSK-effective gene *Sr1RS*^*Amigo*^ [72], though located on 1A, is qualitative in nature and not expected to be present in our panel based on the results from TTKSK screening. Similarly, QTL and genetic markers on 1B that are significantly associated with stem rust resistance have been reported in a durum wheat GWAS study [71], and spring wheat RIL populations [64, 73]. The genomic regions spanned by the QTL in these three chromosomes ranged from 34 Mb (*QSr*.*umn-1D*.*2*) to 184 Mb (*QSr*.*umn-1A*.*1*).

Five, seven, and one QTL were detected on chromosomes 2A, 2B, and 2D, respectively. Three of the seven QTL on 2B lied within < 5 Mb physical distance, with one QTL spanning only about 600 Kb. Several QTL conferring APR to *Pgt* races, including the Ug99 lineage races, on chromosomes 2A, 2B, and 2D have been detected in spring wheat as well as durum wheat mapping populations and genome-wide association study (GWAS) panels [72, 74–76]. The genes *Sr32* (introgressed to 2A, 2B, and 2D), *Sr9h*, *Sr28*, *Sr36*, *Sr39*, *Sr40*, *Sr47* (located on 2B) are effective to races in the Ug99 race group, but are unlikely to exist in our population based on screening for seedling resistance to race TTKSK. Several other genes such as *Sr9a*, *9b*, *9d*, *9e* (located on 2BL); *Sr21* (located on 2AL), and *Sr38* (located on 2AS) are ineffective against African races but are resistant to one or more N. American *Pgt* races [77, 78]. The QTL *QSr*.*umn-2A*.*5* is located only 600 Kb from the distal end of 2AL, yet no known *Sr* genes or QTL are known to exist on this end of 2A. *Sr36*, also located on 2B, is absent in our panel based on marker screening (S1 File).

While 3A harbored four QTL in each of the four environments, 3B had six QTL of which five were common in at least two environments; two QTL were detected on 3D in Ken13 and StP13 environments. The APR gene *Sr2*, located on 3BS, is segregating in four populations: LMPG-6/Gem, LMPG-6/Kudu, LMPG-6/Paka, and LMPG-6/Romany. The presence of *Sr2* in ‘Gem’, ‘Kudu’, ‘Paka’, and ‘Romany’ was confirmed by marker screening (S1 File) as well as expression of the trait pseudo-black chaff (PBC) in StP12 and Ken13 environments on plant internodes (not observed in Romany). PBC is conditioned by the expression of the partially dominant gene *Pbc* causing dark coloring of the glumes and inter-nodal regions in an adult wheat plants, and is considered to be associated with *Sr2* [79, 80]. The physical length of 3B used to map our sequences is 774 Mb and QTL were detected as early as around 69 cM up to 113 cM on distal 3BS. The gene *Sr12* [3], as well as several other QTL identified by mapping studies [64, 71, 81] that confer resistance to stem rust of wheat also exist on 3B. Hence, these QTL could potentially resemble *Sr12* or *Sr28*, of which the latter was absent in our marker screening (S1 File) and the former does not have diagnostic markers available. *Sr51*, a new gene resistant to Ug99, is located on distal end of 3DS and *QSr*.*umn-3D*.*2* is also located approximately 7 Mb from the distal end of 3DS. However, *Sr51* has been introgressed into bread wheat from *Aegilops searsii* and is not currently used in agriculture [72, 82]. *QSr*.*umn-3D*.*2* may represent an ortholog of *Sr51* in bread wheat, or could be a new gene.

We detected three QTL on 4A in Ken13, StP12, and StP13; three on 4B; and one on 4D. Chromosome 4A contains the genes *Sr7a* and *7b*, and chromosome 4B hosts the gene *Sr37* introgressed from *Triticum timopheevi*. *Sr37* is resistant to the Ug99 races, and therefore is unlikely to be present in our population. Both *Sr7a* and *Sr7b* are resistant to multiple N. American *Pgt* races [77, 83]. *Sr7a* was first identified in several Kenyan wheat lines, [67, 68]; and ‘Ngiri’ could have acquired this gene from the line ‘Manitou’ [3], which is present in its pedigree. Thus, this gene may have been bred into one or more of the Kenyan varieties used to create our NAM population. Further screening of the population, preferably in single rust race nurseries, is needed to confirm this postulation. Likewise, the stem rust APR gene *Sr55* (*Lr67/Yr46/Pm46*) is located on 4DL. The only QTL detected on 4D (*QSr*.*umn-4D*) lies on the short arm of 4D (21 cM on linkage group; between 58–61 Mb on 4D which is 121 Mb long) and therefore may not represent *Sr55*. The use of markers *cfd23* and *cfd71* to screen for *Lr67/Yr46/ Sr55*/*Pm46* was inconclusive.

Four QTL were detected on group 5 of chromosomes: two on 5A, one on 5B, and three on 5D of which *QSr*.*umn-5D*.*1* was common in StP12, StP13, and Ken13 environments, and could be involved in broad-spectrum resistance. Several QTL on chromosome 5A that provide resistance against stem rust have been reported in biparental and association mapping studies [60, 64, 71, 73], although no known *Sr* gene has been mapped to either 5A. Chromosome 5B contains *Sr56*, another stem rust APR gene, on its long arm [17]. No diagnostic markers are available to test presence/absence of this gene. Chromosome 5D houses the *Sr* genes *Sr30*, which is known to exhibit resistance to N. American *Pgt* races [57, 72]; and *Sr53*, which is a recent translocation from *Aegilops geniculata* [84], and is not expected to be present in our NAM panel.

On chromosome 6A, three QTL were detected in three different environments; four on 6B (of which *QSr*.*umn-6B*.*2* was common between Ken13 and StP12; and *QSr*.*umn-6B*.*4* was common between Ken13, StP12, and StP13); chromosome 6D was the only chromosome with no QTL detected. Located on 6A are the genes *Sr26* introgressed to common wheat [85, 86]; and *Sr13* in durum wheat (*Triticum durum* Desf.) [71, 87]. *Sr26* is effective against all races of the Ug99 race group [57]. *Sr13* is not prevalent in common wheat [3, 72]. Chromosome 6A also contains the gene *Sr52*, a TTKSK resistant gene recently introgressed into hexaploid wheat from its diploid relative *Dasypyrum villosum* [88]. Hence, it is unlikely that any of these three genes is present in our population. The *Sr* gene *Sr11* is located on 6B [3], in addition to several QTL effective to stem rust on 6A and 6B [60, 64, 71, 86].

*QSr*.*umn-7A*, the only QTL on 7A, is located at the very beginning (3 cM) of the linkage group 7A as well as on the physical map (ranging 2–6 Mb). This is strikingly similar to previous QTL reported on 7AS by Bajgain et al. [75] and Singh et al. [65], and could represent the same locus. Chromosome 7B also had only one QTL detected (*QSr*.*umn-7B*) which was common in all environments except SA12, and may be an important QTL because of its effectiveness in multiple environments. These QTL, located on the short arm, explained 8%—16% of phenotypic variance. The only gene mapped to 7B is *Sr17*, which is ineffective to races of the Ug99 lineage as well as to the N. American *Pgt* races TTTTF, QFCSC, and MCCFC used in this study [77]. Previously reported QTL on chromosome 7B that confer resistance to the Ug99 group of races include the QTL linked to the microsatellite markers *wPt-0318* [60] and *cfa-2040* [71]. The two QTL detected on 7D were mapped close to each other (*QSr*.*umn-7D*.*1* at 25 cM and *QSr*.*umn-7D*.*2* at 26 cM; 16 Mb apart on physical distance) albeit in two different environments. *Sr25*, *Sr43*, and *Sr44* provide resistance to different races within the Ug99 group and are all located on 7DL. Marker screening for *Sr25* showed that the gene is absent in our panel, whereas *Sr43* and *Sr44* are recently introgressed segments from *Thinopyrum elongatum* and *Thinopyrum intermedium*, respectively, and are not currently in use in wheat breeding [72].

Our study uses *de novo* SNP markers, compared to other studies that use microsatellite or chip-based SNP markers. Hence, a direct comparison for names and positions of markers linked to previously reported *Sr* genes and QTL is rather difficult to conduct. As indicated above, the lack of sequence alignment among our SNP markers and published SNP assays prevented from making position-based definitive comparisons with previously detected QTL. We expect that additional research, either jointly or in individual populations, will help in further understanding of the relationship between the discussed *Sr* genes and previously reported QTL with the QTL detected in our study. In particular, construction of a consensus map with previous marker types (SSR, DArT) anchored to the physical map is rather essential to establish a clear connection between sequence-based studies and older studies. Additionally, publication of the wheat genome [45, 46] followed by functional annotation and construction of high density genomic maps can be expected to aid in such comparison, and also in identification of regions with candidate-genes.

## Conclusions

In this study, we present several QTL conferring resistance to the *Pgt* races in a joint mapping approach by using one common parent as a uniform genetic background. The QTL ranged in their allelic effects with small to intermediate contribution in resistance to stem rust of wheat. Epistatic effects among the detected loci did not have significant contributions to resistance, suggesting that the differences between the populations in different environments are mainly due to additive effects of several QTL. QTL common in multiple environments could be used to breed for broad resistance to multiple stem rust races, including the widely virulent African *Pgt* races. Also, by sequencing complexity-reduced genomes of individuals in the NAM panel, we obtained population-specific SNPs that were aligned with the reference genome sequence, and show their usability in mapping QTL. While validation of the detected loci is required to confirm the significant markers, the GBS method could be an alternative for efficient and economical approach for genome mapping and genome-wide studies. In particular, our methodology of reference-based SNP calling followed by QTL mapping confirm that this approach can be used to obtain a higher number of markers to better resolve the D genome. In case of complications that could arise from having undesired amount of missing data, multiple rounds of sequencing or multiplexing with fewer samples could help to obtain enough reads to minimize the problems.

## Supporting Information

S1 Fig Schematic workflow of the study. (PDF)

S2 Fig Principal component analysis of the 10 RIL populations. (PDF)

S3 Fig Principal component analysis of the 11 parents. (PNG)

S4 Fig Distribution of disease severity (%) in the populations. (PDF)

S5 Fig GGE-biplot showing ranking of the lines in four environments. (PNG)

S1 File Spreadsheet containing results data. (XLSX)

S2 File Genotype File Part I, non imputed. (TXT)

S3 File Genotype File Part II, non imputed. (TXT)

S4 File Genotype File Part III, non imputed. (TXT)

S5 File Genotype File Part I, imputed. (TXT)

S6 File Genotype File Part II, imputed. (TXT)

S7 File Genotype File Part III, imputed. (TXT)

S1 Text Comparison between JICIM and ICIM results. (DOCX)
