# Supplementary material for: Nested Association Mapping of Stem Rust Resistance in Wheat Using Genotyping by Sequencing
Source: PLoS One. 2016 May 17;11(5):e0155760. doi: 10.1371/journal.pone.0155760 (PMC4870046; doi:10.1371/journal.pone.0155760)
Supplement: S1 Fig — (PDF) [file pone.0155760.s001.pdf]

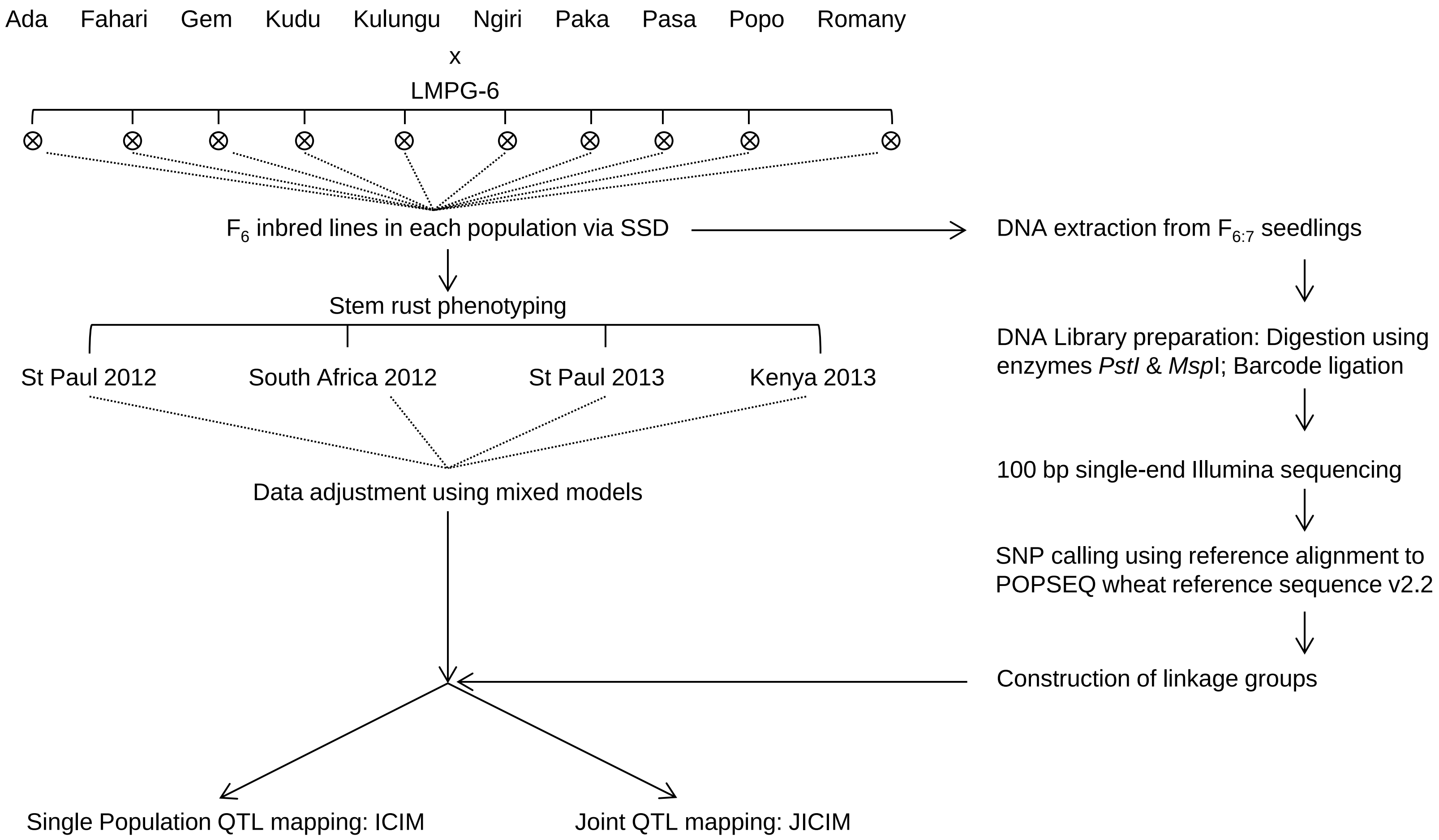

Ada
Fahari
Gem
Kudu
Kulungu
Ngiri
Paka
Pasa
Popo
Romany
x
LMPG-6
F_6 inbred lines in each population via SSD
DNA extraction from $F_{6:7}$ seedlings
Stem rust phenotyping
St Paul 2012
South Africa 2012
St Paul 2013
Kenya 2013
DNA Library preparation: Digestion using enzymes *PstI* & *Msp*I; Barcode ligation
100 bp single-end Illumina sequencing
Data adjustment using mixed models
SNP calling using reference alignment to POPSEQ wheat reference sequence v2.2
Construction of linkage groups
Single Population QTL mapping: ICIM
Joint QTL mapping: JICIM
