# Supplementary figures and images for: Nested Association Mapping of Stem Rust Resistance in Wheat Using Genotyping by Sequencing
Source: PLoS One. 2016 May 17;11(5):e0155760. doi: 10.1371/journal.pone.0155760 (PMC4870046; doi:10.1371/journal.pone.0155760)

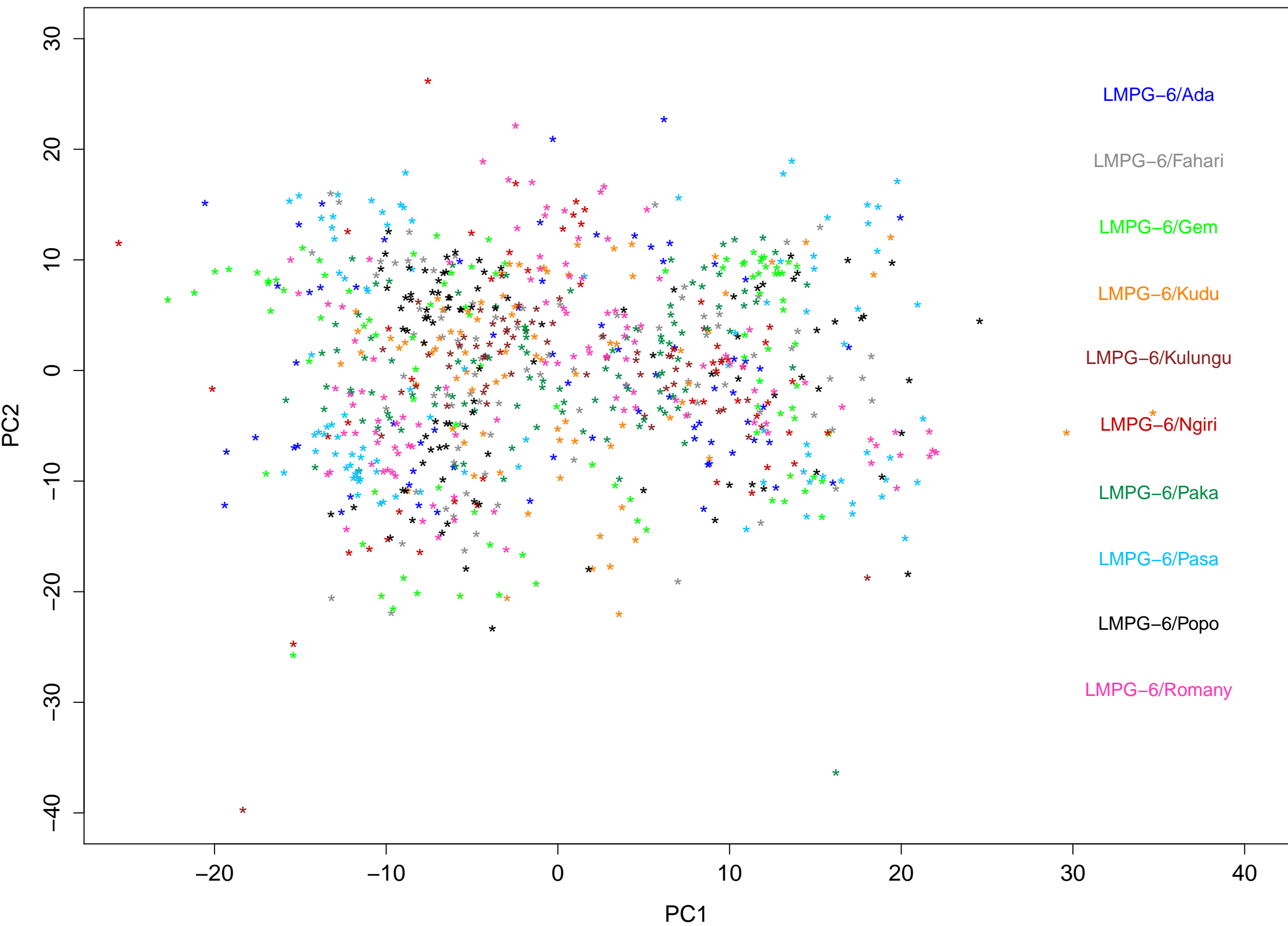

Supplement: S2 Fig — (PDF) [file pone.0155760.s002.pdf]

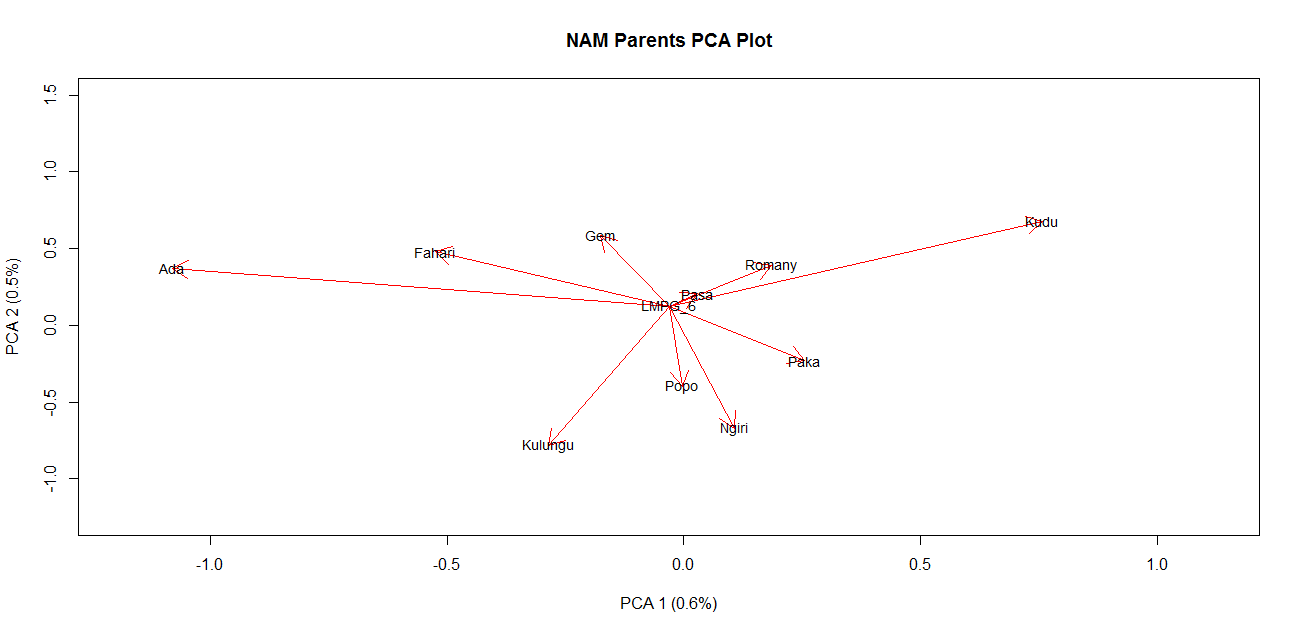

Supplement: S3 Fig — (PNG) [file pone.0155760.s003.png]

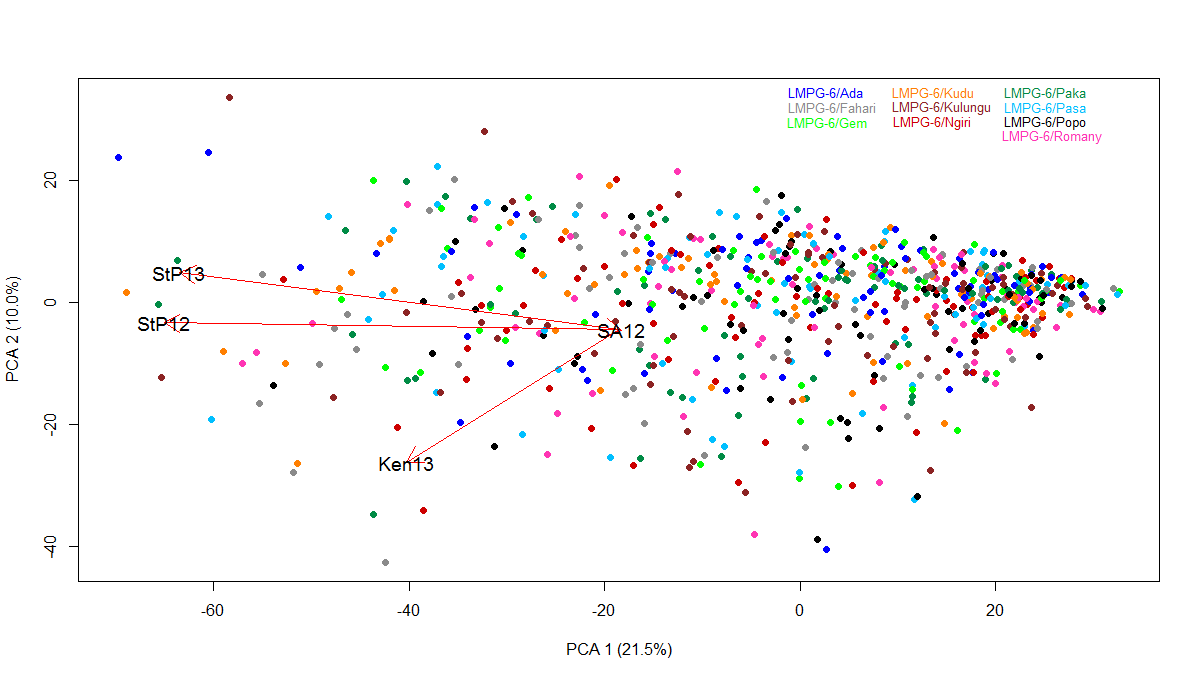

Supplement: S5 Fig — (PNG) [file pone.0155760.s005.png]
