# Supplementary material for: Nested Association Mapping of Stem Rust Resistance in Wheat Using Genotyping by Sequencing
Source: PLoS One. 2016 May 17;11(5):e0155760. doi: 10.1371/journal.pone.0155760 (PMC4870046; doi:10.1371/journal.pone.0155760)
Supplement: S4 Fig — (PDF) [file pone.0155760.s004.pdf]

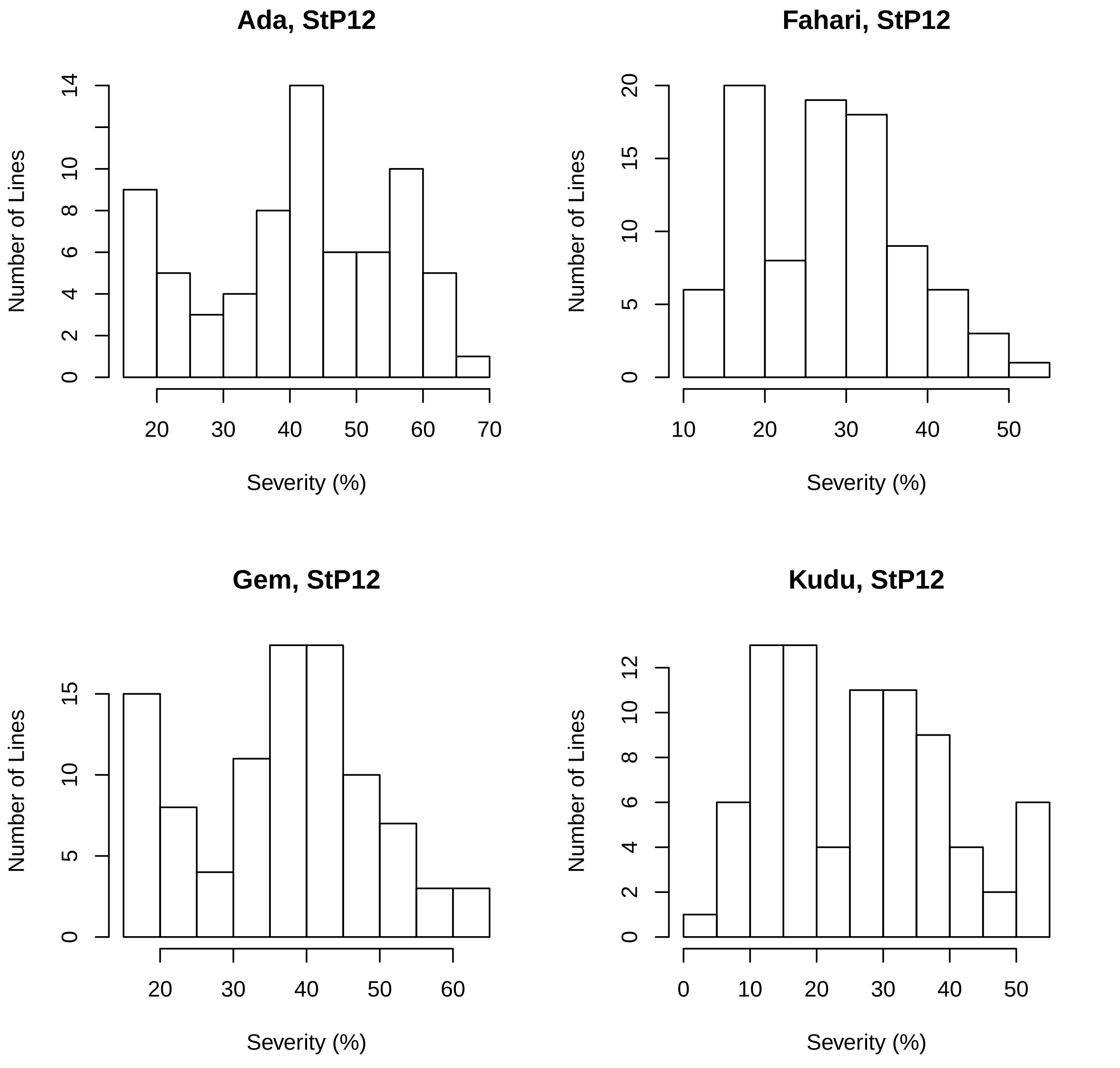
Ada, StP12
Number of Lines
0 2 4 6 8 10 14
20 30 40 50 60 70
Severity (%)
Fahari, StP12
Number of Lines
0 5 10 15 20
10 20 30 40 50
Severity (%)
Gem, StP12
Number of Lines
0 5 10 15
20 30 40 50 60
Severity (%)
Kudu, StP12
Number of Lines
0 2 4 6 8 10 12
0 10 20 30 40 50
Severity (%)

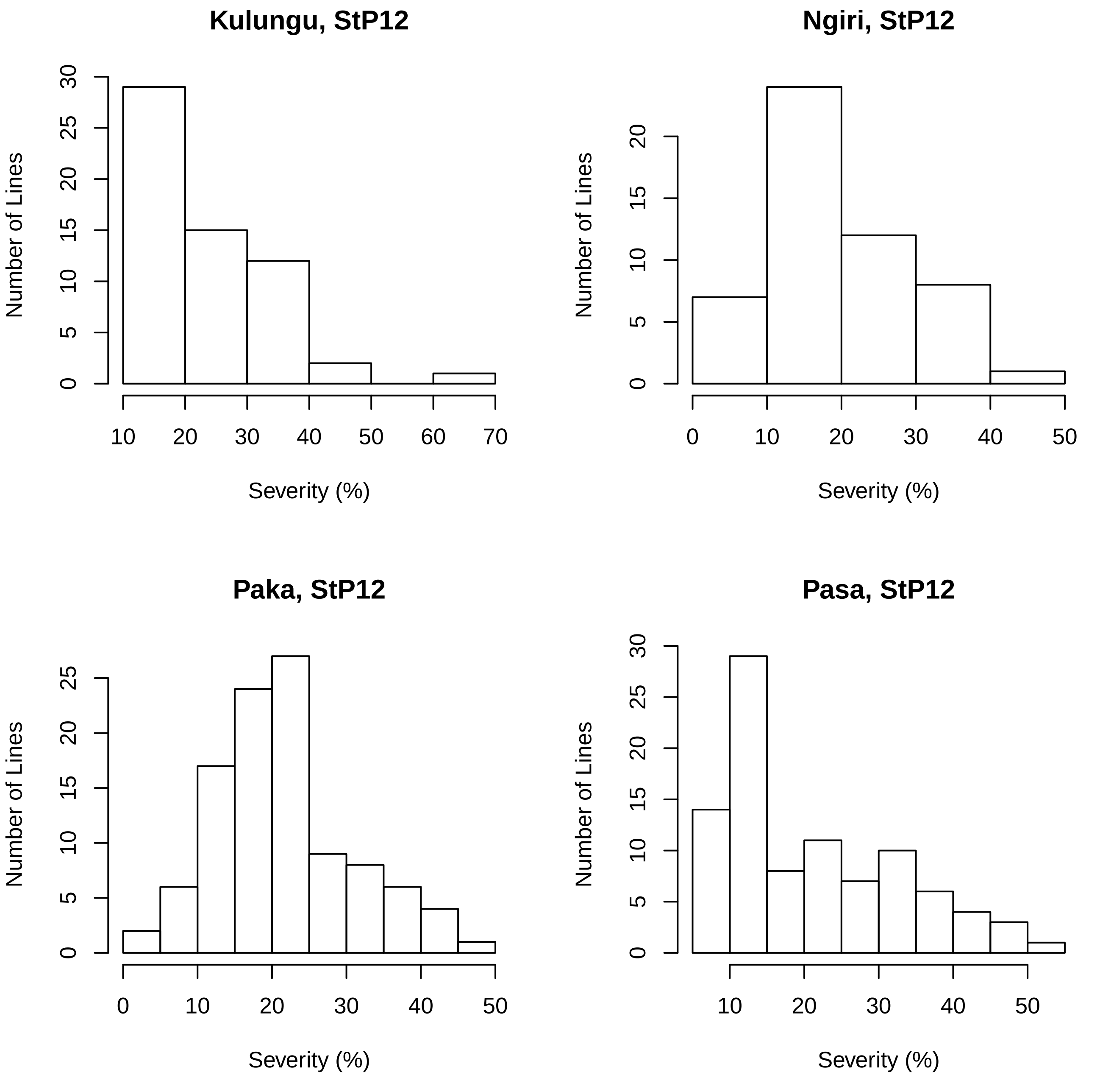
Kulungu, StP12
Number of Lines
0
5
10
15
20
25
30
10
20
30
40
50
60
70
Severity (%)
Ngiri, StP12
Number of Lines
0
5
10
15
20
0
10
20
30
40
50
Severity (%)
Paka, StP12
Number of Lines
0
5
10
15
20
25
0
10
20
30
40
50
Severity (%)
Pasa, StP12
Number of Lines
0
5
10
15
20
25
30
10
20
30
40
50
Severity (%)

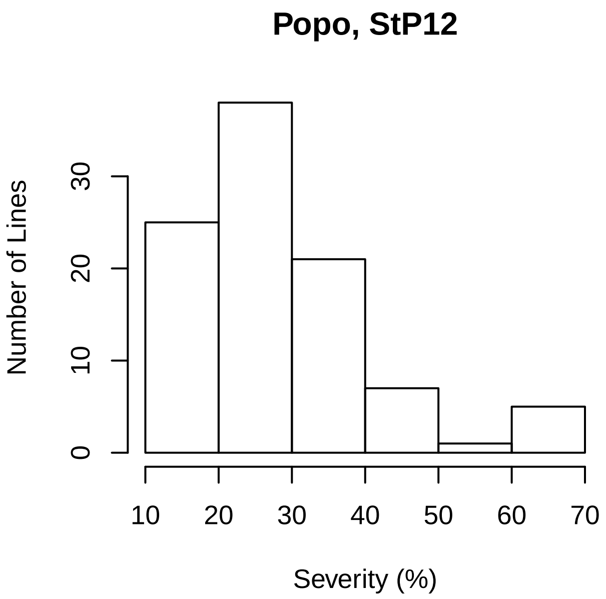

Popo, StP12
Number of Lines
0
10
20
30
10
20
30
40
50
60
70
Severity (%)

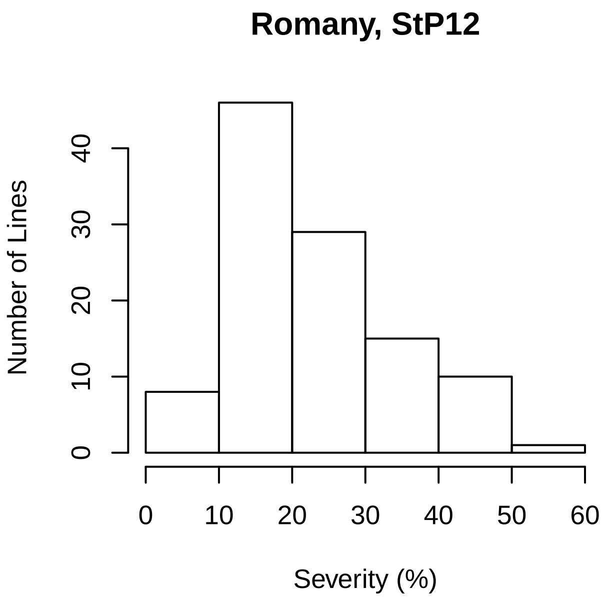

Romany, StP12
Number of Lines
0
10
20
30
40
0
10
20
30
40
50
60
Severity (%)

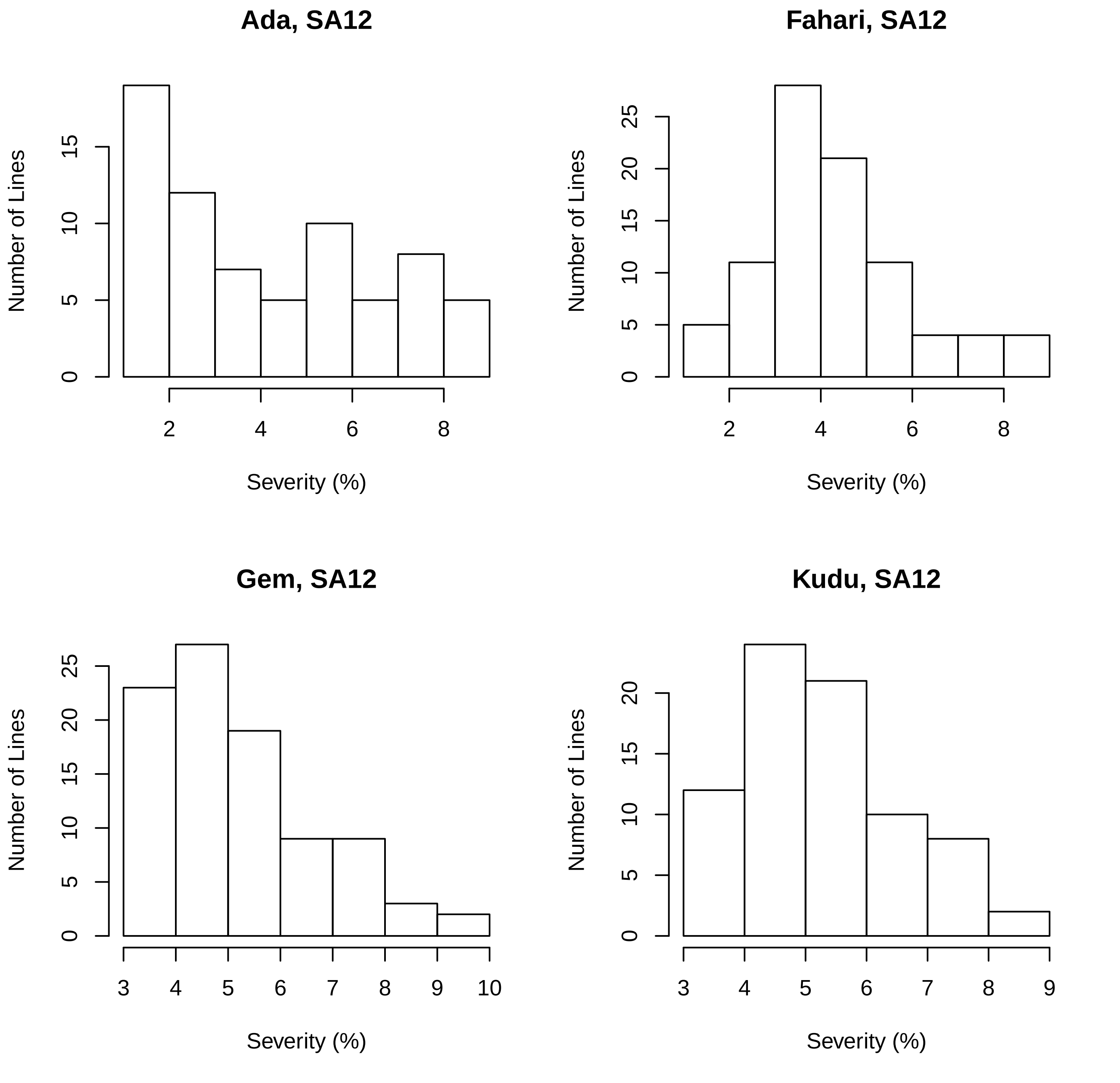
Ada, SA12
Number of Lines
0
5
10
15
2
4
6
8
Severity (%)
Fahari, SA12
Number of Lines
0
5
10
15
20
25
2
4
6
8
Severity (%)
Gem, SA12
Number of Lines
0
5
10
15
20
25
3
4
5
6
7
8
9
10
Severity (%)
Kudu, SA12
Number of Lines
0
5
10
15
20
3
4
5
6
7
8
9
Severity (%)

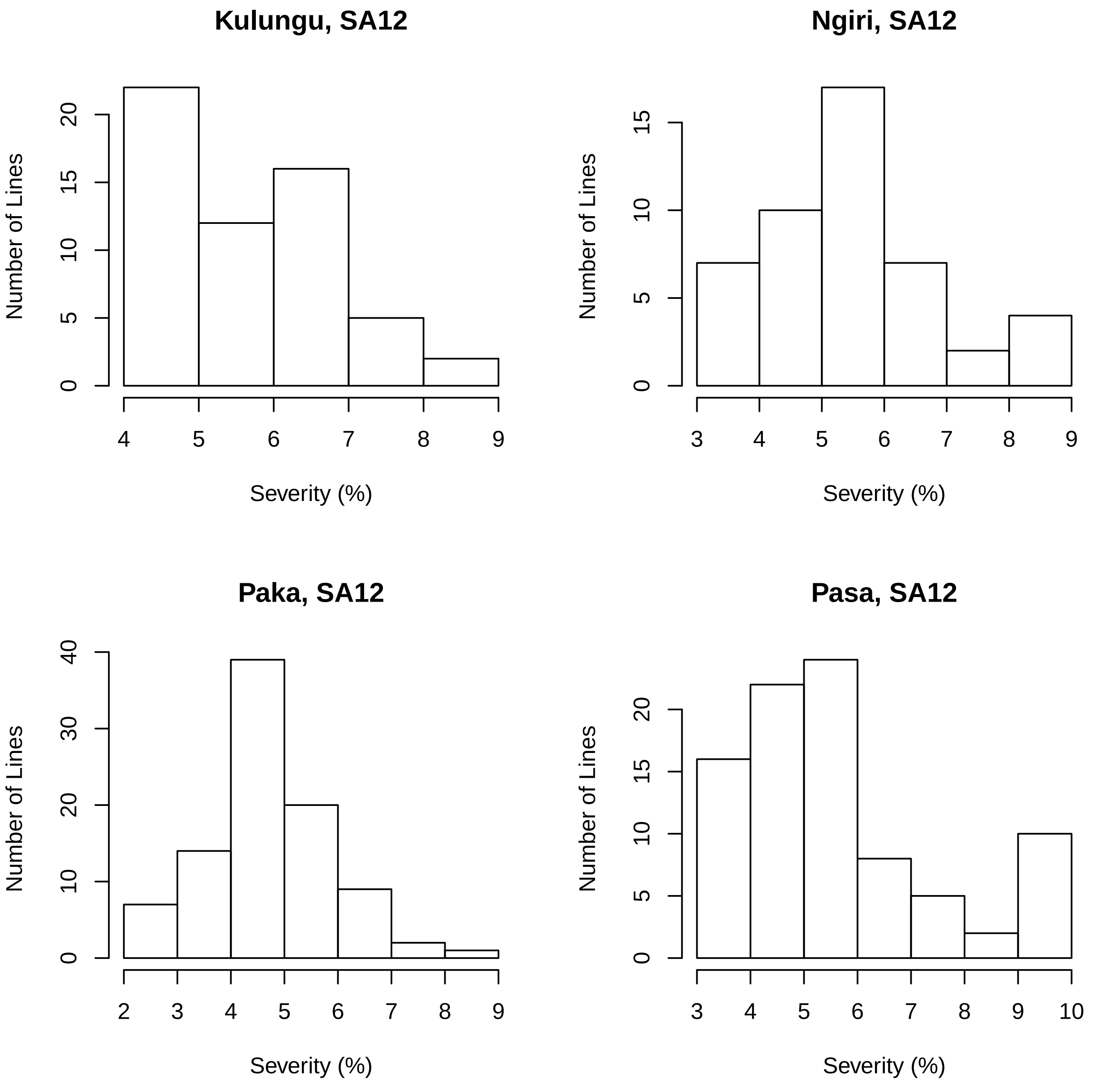

Kulungu, SA12
Number of Lines
0
5
10
15
20
4
5
6
7
8
9
Severity (%)
Ngiri, SA12
Number of Lines
0
5
10
15
3
4
5
6
7
8
9
Severity (%)
Paka, SA12
Number of Lines
0
10
20
30
40
2
3
4
5
6
7
8
9
Severity (%)
Pasa, SA12
Number of Lines
0
5
10
15
20
3
4
5
6
7
8
9
10
Severity (%)

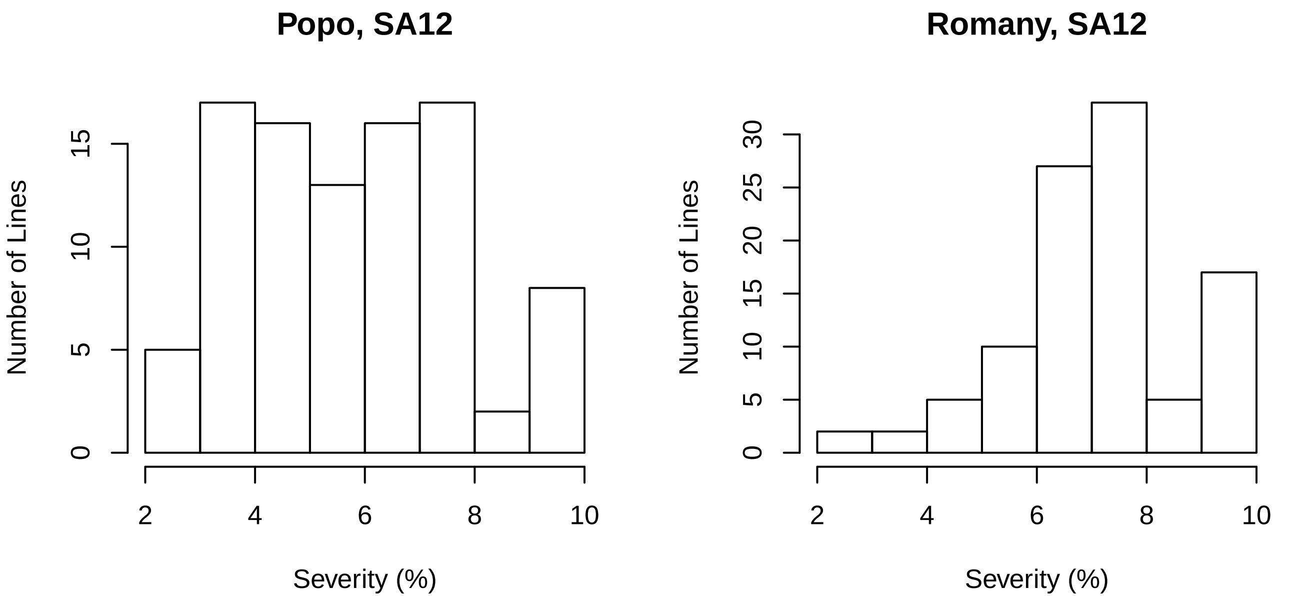
Popo, SA12
Number of Lines
0
5
10
15
2
4
6
8
10
Severity (%)
Romany, SA12
Number of Lines
0
5
10
15
20
25
30
2
4
6
8
10
Severity (%)

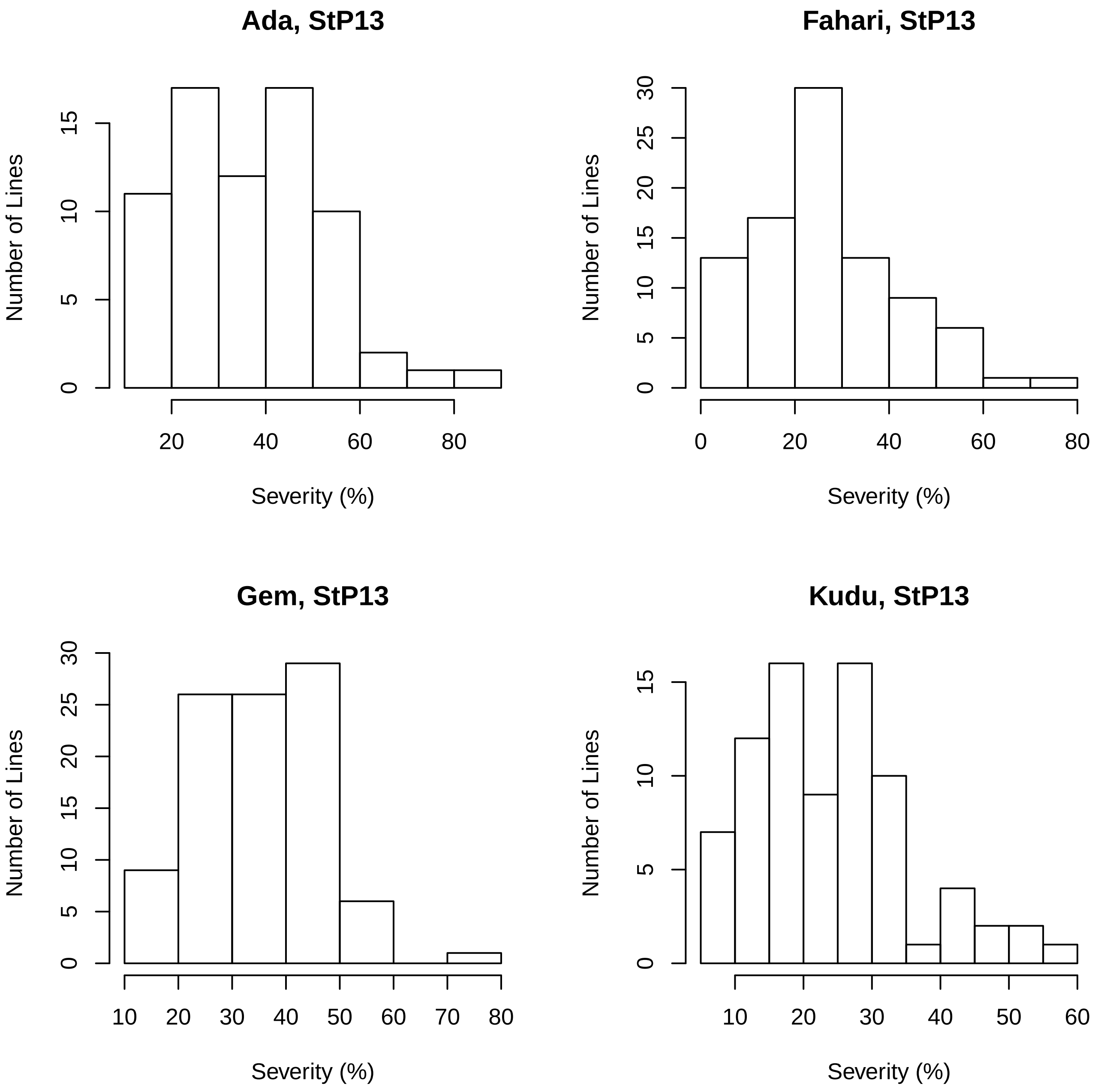
Ada, StP13
Number of Lines
Severity (%)
Fahari, StP13
Number of Lines
Severity (%)
Gem, StP13
Number of Lines
Severity (%)
Kudu, StP13
Number of Lines
Severity (%)

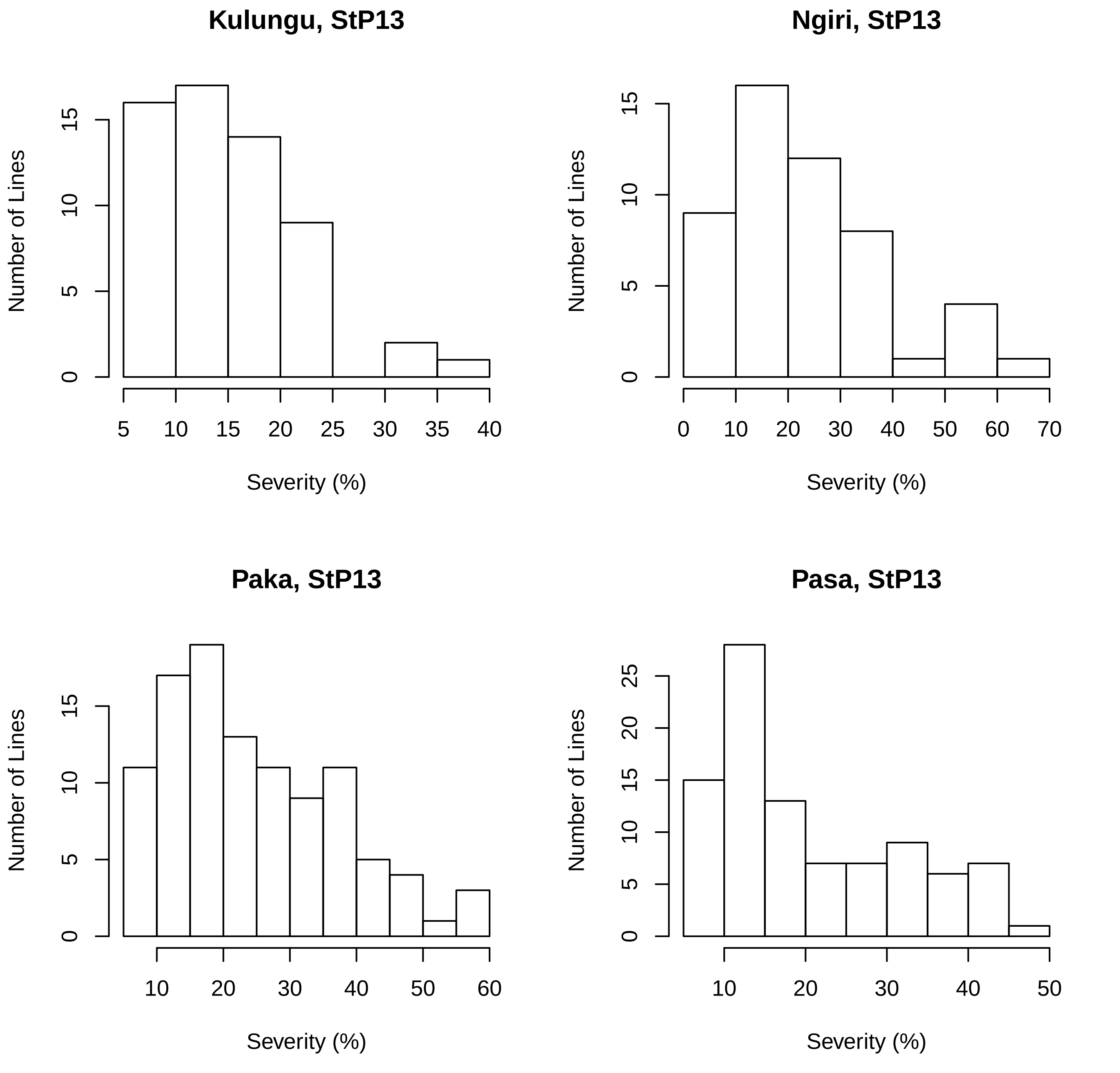

Kulungu, StP13
Number of Lines
0
5
10
15
5
10
15
20
25
30
35
40
Severity (%)
Ngiri, StP13
Number of Lines
0
5
10
15
0
10
20
30
40
50
60
70
Severity (%)
Paka, StP13
Number of Lines
0
5
10
15
10
20
30
40
50
60
Severity (%)
Pasa, StP13
Number of Lines
0
5
10
15
20
25
10
20
30
40
50
Severity (%)

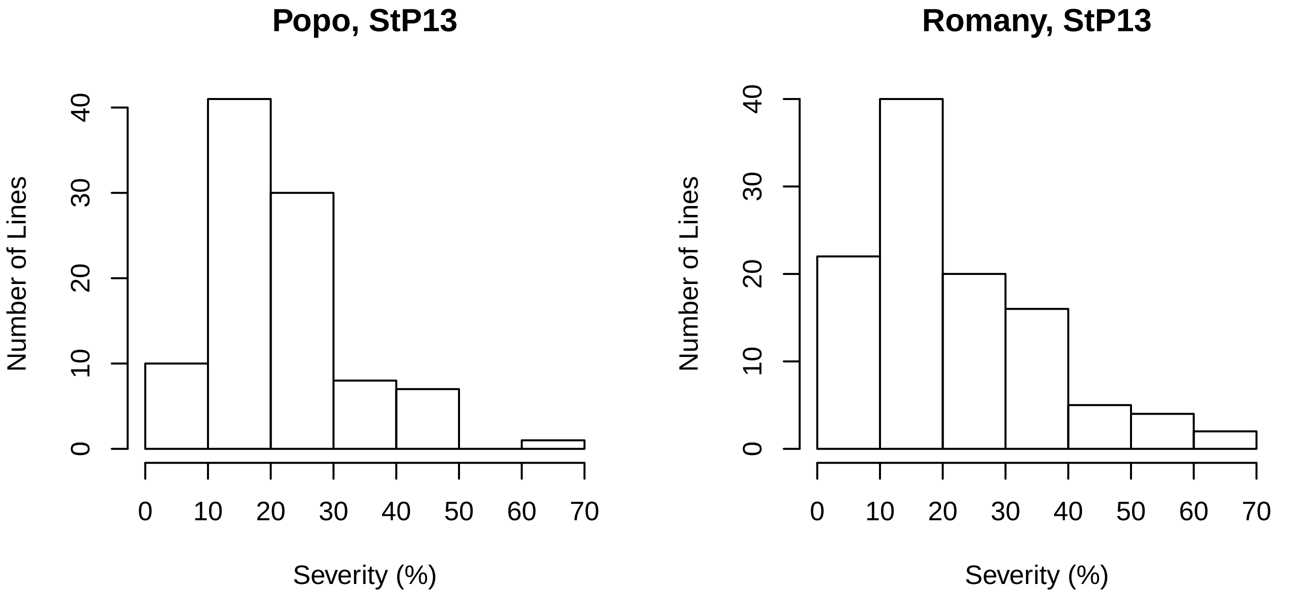

Popo, StP13
Number of Lines
0
10
20
30
40
0
10
20
30
40
50
60
70
Severity (%)
Romany, StP13
Number of Lines
0
10
20
30
40
0
10
20
30
40
50
60
70
Severity (%)

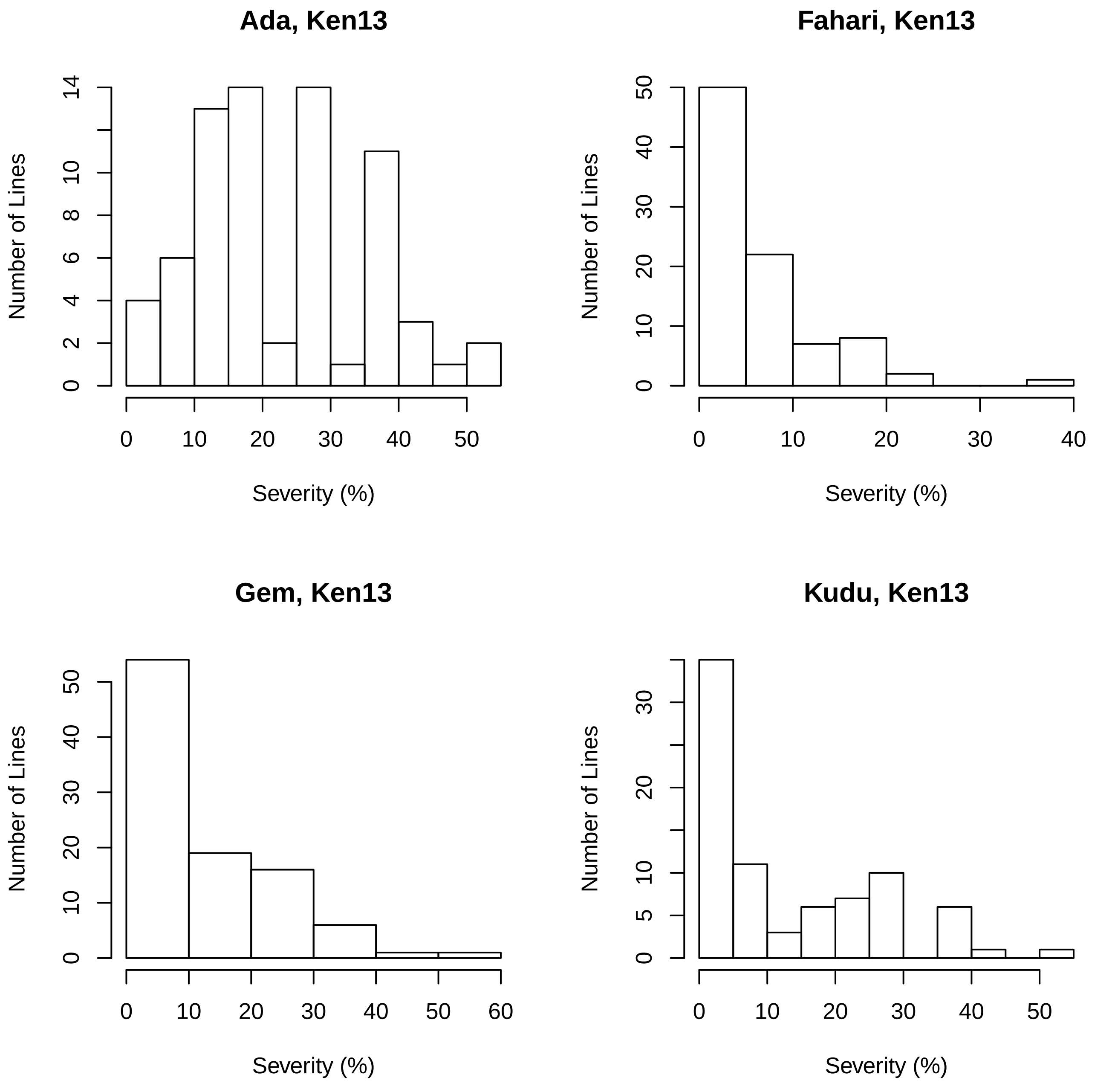
Ada, Ken13
Fahari, Ken13
Gem, Ken13
Kudu, Ken13
Number of Lines
Severity (%)

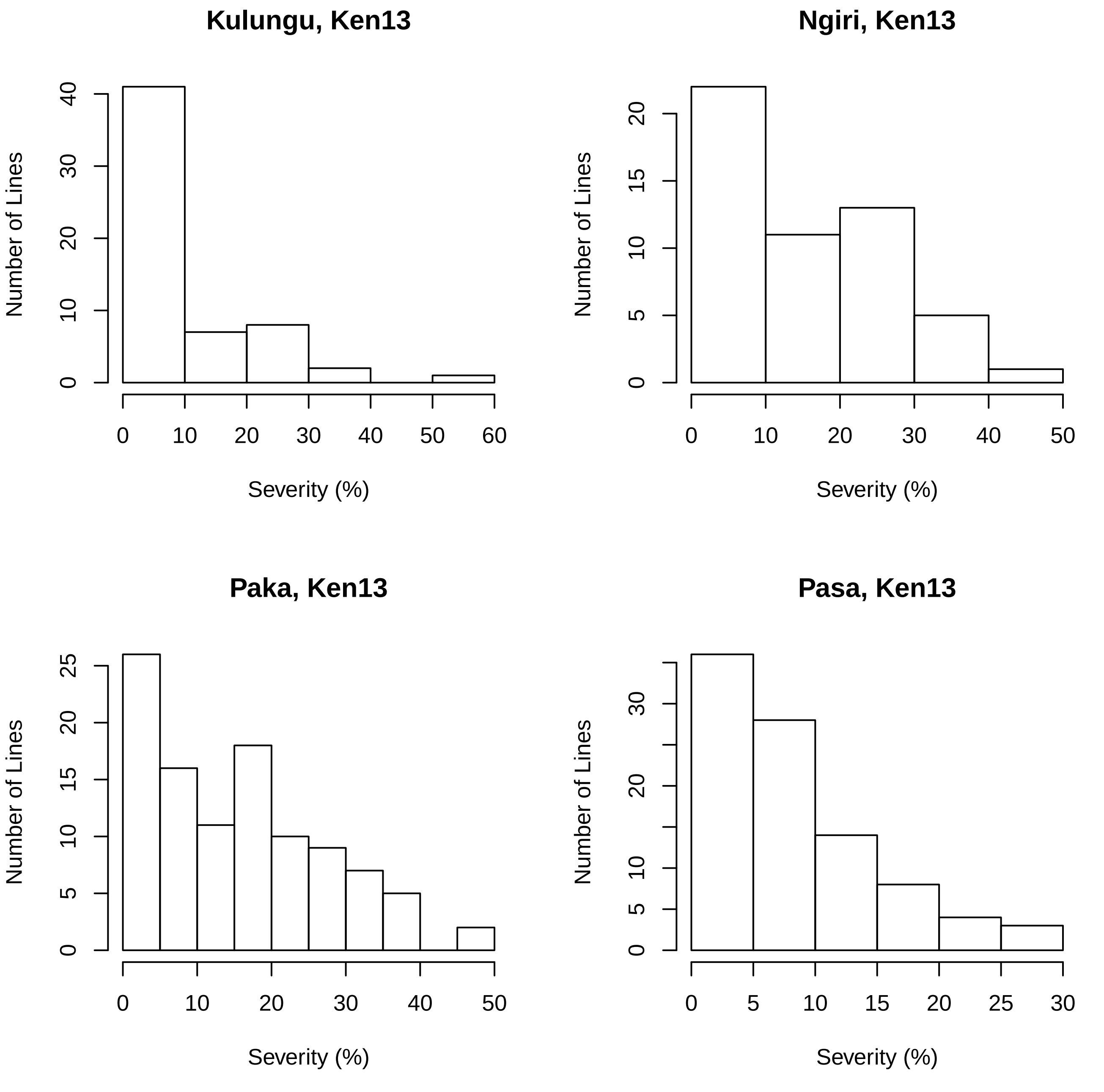
Kulungu, Ken13
Number of Lines
Severity (%)
0
10
20
30
40
0
10
20
30
40
50
60
Ngiri, Ken13
Number of Lines
Severity (%)
0
5
10
15
20
0
10
20
30
40
50
Paka, Ken13
Number of Lines
Severity (%)
0
5
10
15
20
25
0
10
20
30
40
50
Pasa, Ken13
Number of Lines
Severity (%)
0
5
10
20
30
0
5
10
15
20
25
30

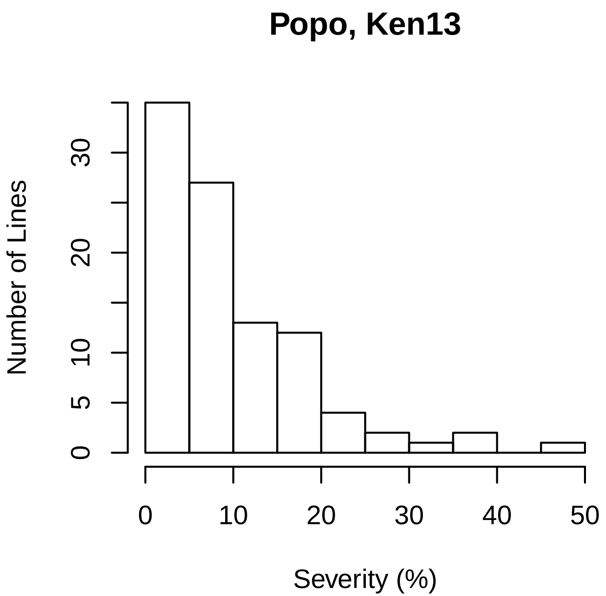
Popo, Ken13
Number of Lines
0
5
10
20
30
0
10
20
30
40
50
Severity (%)

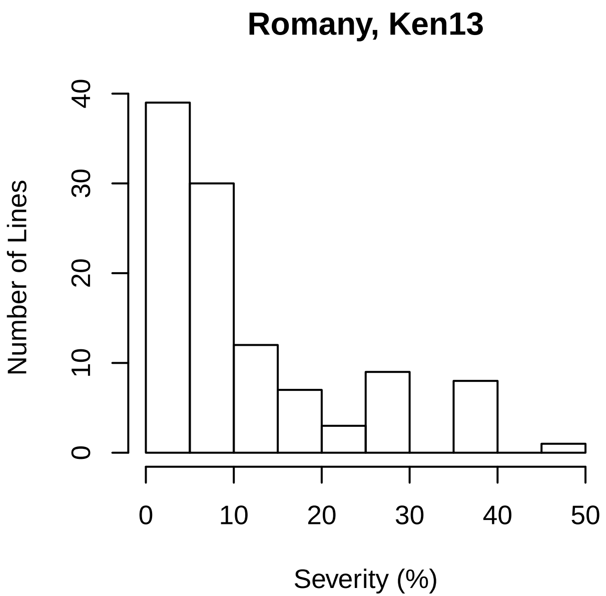
Romany, Ken13
Number of Lines
0
10
20
30
40
0
10
20
30
40
50
Severity (%)
