# Supplementary material for: Nested Association Mapping of Stem Rust Resistance in Wheat Using Genotyping by Sequencing
Source: PLoS One. 2016 May 17;11(5):e0155760. doi: 10.1371/journal.pone.0155760 (PMC4870046; doi:10.1371/journal.pone.0155760)
Supplement: S1 Text — (DOCX) [file pone.0155760.s013.docx]

**Comparison between JICIM and ICIM results**

The JICIM method detected QTL in all environments and on all chromosomes (except 6D) where QTL were detected by ICIM in each population. The environments with the most QTL detected were StP13 in JICIM (26) and StP12 in ICIM (21), whereas SA12 recorded the least number of QTL (8) in JICIM and StP13 (10) in ICIM. While the additive effects contributed by the parents were similar between the two methods, the percent of phenotypic variation (R^2^) explained by the QTL were strikingly different. The R^2^ value of QTL detected in JICIM ranged from 1.4% to 20.4% whereas it ranged from 1.4% to 76.0% in ICIM. As QTL in joint mapping are declared based on their overall contribution to resistance (or susceptibility) across all 10 families, a ‘normalized’ value is calculated with the effect of each parent taken into account. In other words, a joint analysis estimates the allelic effects of a QTL by assuming that the allele from a founder parent is uniform across all populations ([Rebai and Goffinet 1993](#_ENREF_2); [Blanc et al. 2006](#_ENREF_1)). This might not necessarily be true for each QTL detected, especially in case of linked genes, and among populations that differ in the number and effect of loci associated with the trait. In our study, via ICIM of single populations, we have found that not all populations share the same locus for stem rust resistance (Supplemental File 1). Hence, such an assumption in the joint mapping model could be one of the main reasons why the joint mapping differs from individual mapping in estimation of the amount of total phenotypic variance accounted by the significant SNPs. Validation of the QTL detected in both approaches will help to further elucidate the relationship between the QTL detected in these two mapping approaches.

**References**

Blanc G, Charcosset A, Mangin B, Gallais A, Moreau L (2006) Connected populations for detecting quantitative trait loci and testing for epistasis: an application in maize. Theor Appl Genet 113:206-224

Rebai A, Goffinet B (1993) Power of tests for QTL detection using replicated progenies derived from a diallel cross. Theor Appl Genet 86:1014-1022
